# MMP7 cleavage of amino-terminal CD95 death receptor switches signaling toward non-apoptotic pathways

**DOI:** 10.1038/s41419-022-05352-0

**Published:** 2022-10-23

**Authors:** Shoji F. Kenji, Keerthi Kurma, Brigitte Collet, Christelle Oblet, Laure Debure, Carmelo Di Primo, Laëtitia Minder, Franck Vérité, Yannic Danger, Mickael Jean, Aubin Penna, Nicolas Levoin, Patrick Legembre

**Affiliations:** 1 IRSET, INSERM U1085, Université de Rennes 1, 36043 Rennes, France; 2 INSERM U1262, Université de Limoges, 2, Rue Marcland, 87025 Limoges, France; 3 Centre Eugène Marquis, rue bataille Flandres Dunkerque, 35042 Rennes, France; 4 Université de Rennes-1, INSERM U1242, rue bataille Flandres Dunkerque, 35042 Rennes, France; 5 University Bordeaux, CNRS, INSERM, ARNA, UMR 5320, U1212, IECB, F-33000 Bordeaux, France; 6 University Bordeaux, CNRS, INSERM, UAR 3033, US001, IECB, F-33000 Bordeaux, France; 7 EFS Rennes, Rue Pierre Jean Gineste, 35016 Rennes Cedex, France; 8 Université de Rennes 1, Institut des Sciences Chimiques de Rennes - UMR CNRS 6226 Equipe COrInt, F-35000 Rennes, France; 9 4CS, CNRS UMR6041, Université de Poitiers, 86073 Poitiers, France; 10 Bioprojet Biotech, rue du Chesnay Beauregard, 35760 Saint-Grégoire, France

**Keywords:** Protein aggregation, Protein aggregation

## Abstract

CD95 is a death receptor that can promote oncogenesis through molecular mechanisms that are not fully elucidated. Although the mature CD95 membrane receptor is considered to start with the arginine at position 17 after elimination of the signal peptide, this receptor can also be cleaved by MMP7 upstream of its leucine at position 37. This post-translational modification occurs in cancer cells but also in normal cells such as peripheral blood leukocytes. The non-cleaved CD95 amino-terminal region consists in a disordered domain and its in silico reconstitution suggests that it might contribute to receptor aggregation and thereby, regulate the downstream death signaling pathways. In agreement with this molecular modeling analysis, the comparison of CD95-deficient cells reconstituted with full-length or N-terminally truncated CD95 reveals that the loss of the amino-terminal region of CD95 impairs the initial steps of the apoptotic signal while favoring the induction of pro-survival signals, including the PI3K and MAPK pathways.

## Introduction

CD95 (also known as Fas) belongs to the TNF receptor family and its stimulation by its natural ligand, CD95L, is instrumental in tumor surveillance, immune tolerance and homeostasis, as evidenced by clinical symptoms in human patients affected by autoimmune lymphoproliferative syndrome (ALPS) type IA [1]. CD95L is a member of the TNF superfamily. Upon binding of CD95L, the intracellular death domain (DD) of CD95 recruits the adaptor Fas-associated death domain (FADD) protein and the caspase-8 proenzyme, leading to caspase activation and apoptosis [2]. Furthermore, CD95L can be cleaved by metalloprotease to release a soluble ligand that fails to trigger cell death but induces non-apoptotic signaling pathways promoting metastasis in cancers [3, 4] and inflammation [5–8].

CD95 is a 335 amino acid (319 amino acids for the mature protein) type 1 transmembrane glycoprotein containing three cysteine-rich domains (CRDs) in its extracellular region, with CRD2 and the upper part of CRD3 forming the region of interaction with CD95L [9]. The N-terminal region of CRD1 contains a homotypic interaction domain termed pre-ligand assembly domain (PLAD) contributing to the self-association of the receptor [10]. The minimal PLAD region has been precisely mapped to the amino acid residues 59 to 82 [11], raising the question of the importance of the first 58 amino acids for CD95 biological functions.

Using cancer cell lines and blood cells from healthy donors, we observed that all cells, but macrophages, expressed a plasma membrane CD95 truncated in its N-terminal extremity. Home-made monoclonal antibodies and epitope mapping allowed us to establish that CD95 is cleaved between glutamic acid at position 36 and leucine at position 37 (^36^EL^37^) within the PLAD. Finally, we observed that this cleavage impacts receptor aggregation, impairing the induction of the apoptotic response to the benefit of pro-survival signaling pathways.

## Materials and methods

### Antibodies and reagents

Mouse monoclonal anti-FLAG M2 (#F3165) and anti-β-actin (#A5316) antibodies were from sigma-Aldrich. Polyclonal rabbit anti-CD95 antibodies (C20, #SC-715; N18, #SC-714) were from Santa Cruz Biotechnologies. Anti-CD95 (APO1-3, #ALX-805-020) was purchased from Enzo Life Science (Villeurbane, France). Anti-Akt (#CST 9272), anti-Akt-pS473 (D9E, #4060), anti-ERK (#9102) and anti-ERK pT202/204 (197G2, #4377) antibodies were from Cell Signaling Technology (Boston, MA). Mouse purified Anti-HA Tag (#11A3A05) was from Biolegend. Anti-CD19-PE-Cy7 (#560728), anti CD56-PE (#556647), anti CD14-PE (#555398), anti CD203c-APC (#562973), anti SIGLEC5-APC, anti-Rabbit-PE (#558416), anti-Mouse-PE (#550589), anti-FADD (#556402) and anti-CD95L (G247-4, # 556387) were acquired from BD Bioscience. Anti-Rabbit AlexaFluor555 (#A-21428) and Anti-Mouse AlexaFluor555 (#A-21422) were from Thermo Fischer Scientific. Wortmannin and UO126 were purchased from Calbiochem (Merck Chemicals Ltd., Nottingham, UK). DAPI, TMRM (Tetramethyl rhodamine methyl ester perchlorate), MTT (Thiazolyl Blue Tetrazolium Bromide), Duolink starter kit, protease and phosphate cocktails were purchased from Sigma-Aldrich. Recombinant MMP7 and MMP3 were purchased from Merck Millipore (Merck Chimie SAS, Île-de-France, France).

### Plasmid constructs

The multi-aggregated CD95L (Ig-CD95L), membrane wild-type and amino-acid 1-58 truncated CD95 constructs were generated in our laboratory, as described previously (Edmond, 2012). Soluble full-length and 1-58-truncated CD95 constructs were generated by PCR amplifying the CD95 sequences coding for amino-acids 1-158 and 43-158 respectively. For both constructs, PCR primers were designed in order to insert a Kpn1 restriction site followed by the HA signal peptide sequence in 5′ and Flag/hexahistidine epitopes sequences followed by a stop codon and a Xho1 restriction site in 3′. (Wt CD95 forward primer: 5’-cgggtaccatggctatcatctacctcatcctcctgttcaccgctgtgcggggcagattatcgtccaagagtg-3′; 1-58 truncated CD95 forward primer: 5′-cgggtaccatggctatcatctacctcatcctcctgttcaccgctgtgcggggctgccataagccctgtcc-3′; reverse primer: 5′-ccgctcgagctaatggtgatggtgatgatggccgcccttgtcatcgtcgtccttgtagtcgaattccaagttagatctggatccttcc-3′.

PCR products were digested by Kpn1/Xho1 and inserted in the Kpn1/Xho1-linearized pcDNA3.1(+)vector (Invitrogen). All sequences were verified by DNA sequencing on both strand (GATC, Mulhouse, France). Constructs consisting in albumin signal peptide (MKWVTFISLLFLFSSAYS) [12] fused to sCD95-Δ36 (a.a. 37-173) and sCD95-K33G (a.a. 17-173; AAG codon (K) was replaced by GGG (G)) and conjugated in C-term to the FLAG and 6xHis tags were synthesized and cloned (HindIII-EcoRI) in pCDNA3.1(+) (Genecust, Chalmont, France).

### Cells

CEM-IRC (IRC for Ig-CD95L-Resistant cells) cells were obtained as described previously (Edmond, 2012) and maintained in RPMI1640 medium supplemented with 8% v/v heat-inactivated FCS and 2 mM L-glutamine at 37 °C in a 5% CO_2_ incubator. Transfection was done by electroporating cells (0.3 ml) with 10 µg of DNA at 200 V/65 ms using a BTM 830 electroporation generator (BTX, Holliston, MA). Transfected CEM-IRC cells were selected using medium supplemented with 0.8 mg/ml of neomycin. Clones were obtained by the limiting dilution technique and CD95 plasma membrane expression was assessed by flow cytometry.

The haematological cell lines SKW6.4, H9, CEM, JURKAT, RPMI1826, K562, MOLT-4, CCR-CEM; breast cancer cell lines MCF-7, T47D, ZR75, BT474, MDA-MB453, MDA-MB-231, MDA-MB-468, BT549, HS578T, HCT-116, colon cancer cell lines HCT-116, SW620, HCT-15, KM12, HCCT2958, COLO205, HT29, renal cell lines ACH, 786-0, UO-31; glioblastoma cell line SNB-19; ovarian cell line SK-OV3; prostate cell line DUO-147 and melanoma cell lines, MALME-3M and UACC257 were obtained from the National Cancer Institute. PBLs were obtained after Ficoll centrifugation and removal of blood erythrocytes from normal blood donors.

### Cell Death Assays

Cell viability was evaluated using MTT assay, as previously described [13]. Briefly, cells (4.10^4^) were cultured for 24 h in flat-bottom 96-well plates with the indicated concentrations of the apoptosis inducer in a final volume of 100 µl for the time indicated in figure legends. Then, 15 µl of MTT (5 mg/ml in PBS) solution was added during 4 h of incubation at 37 °C and revealed using 105 µl of a solution containing 5% formic acid dissolved in isopropanol. Absorbance was then measured at 570 nm wavelength using the EnSpire^®^ 2300 Multilabel Plate Reader reader (Perkin Elmer, Waltham, Massachusetts, USA). To evaluate early stages of apoptosis, the Tetramethylrhodamine Methyl Ester, Perchlorate (TMRM) was used to determine the drop of ΔΨ_*m*_ as an early event of apoptosis. To this end, cells (10^5^) were incubated with 10 nM TMRM in PBS for 15 min at 37 °C, washed and then stimulated with the IgCD95L (100 ng/ml) for the indicated times. TMRM fluorescence was detected in the FL-2 channel (560 nm) of the flow cytometer and data analyzed by Cell Quest software (BD bioscience, San Jose, CA, USA).

### Immunoblotting

Western-blots of total cell lysates were performed as previously described [7]. Briefly, cells were washed once with ice-cold PBS and permeabilized for 30 min on ice using a HEPES-Triton X-100 based lysis buffer (25 mM HEPES pH 7.4, 1% (v/v) Triton X-100, 150 mM NaCl, and 2 mM EGTA supplemented with protease and phosphatase inhibitor cocktail (Sigma)). Protein concentration was determined by the bicinchoninic acid method (Pierce, Rockford, IL, USA) according to the manufacturer’s protocol. A total of 50 to 100 μg of protein was loaded per lane, resolved by SDS-PAGE gel and transferred to a nitrocellulose membrane (GE Healthcare, Buckinghamshire, UK). Non-specific binding sites were blocked by incubating membranes for 30 min with TBST-BSA (50 mM Tris, 160 mM NaCl, 0.05% (v/v) Tween 20, and 5% BSA at pH 7.4) or TBST-milk (50 mM Tris, 160 mM NaCl, 0.05% (v/v) Tween 20 and 5% (w/v) dried skimmed milk at pH 7.4) and later incubated overnight at 4 °C with the corresponding primary antibody. For visualization of bound primary antibodies, membranes were washed with TBST, incubated with an appropriate peroxidase-labeled antibody for 45 min and then visualized using an enhanced chemiluminescence substrate kit (ECL RevelBot Ozyme, Saint Quentin en Yvelines, France) according to the protocols delivered by the manufacturers.

### Secreted CD95 constructs production

Full-length extracellular domain of CD95 (sCD95 for soluble CD95), K33G (sCD95-K33G) mutant and Δ36- or Δ58-truncated counterparts (sCD95-Δ36 and sCD95-Δ58)-encoding vectors were transfected using calcium/phosphate method in HEK/293 T cells. After 7 days of culture in serum-deprived Opti-MEM (Life technologies, Saint Aubin, France), secreted constructs were collected. Supernatants were ultracentrifuged for 3 h (100 000 g) to eliminate micro-vesicle contamination. Soluble CD95 concentration was quantified by ELISA (Abcam, MA, USA).

### Gel Filtration

The native molecular weight of sCD95 or sCD95-Δ58 was assessed by gel filtration using Sephacryl S-200 High Resolution columns (GE Healthcare) equilibrated with PBS. Using an AKTAprime plus apparatus (GE Healthcare), proteins were eluted at a flow rate of 0.5 mL/min and fractions (2 ml) were harvested. The distribution of sCD95 and sCD95-Δ58 was next revealed by 12% SDS-PAGE. The native molecular weights of sCD95-Δ36 and sCD95-K33G constructs were assessed by gel filtration using a XK 16/70 column filled with Superdex 200 Prep Grade resin (GE Healthcare) and equilibrated with PBS. Using an ÄKTA FPLC™ system (GE Healthcare), proteins were eluted at 1 mL/min and fractions of 1 mL were harvested.

### DISC immunoprecipitations

Cells (2.10^7^) were treated with 100 ng/ml of IgCD95L for the times indicated at 37 °C. Next, cells were incubated for 30 min in a HEPES-Triton X-100 based lysis buffer on ice. CD95 DISC was immunoprecipitated using the APO1-3 antibody for 20 min at 4 °C. Protein A-Sepharose beads (Sigma-Aldrich) were used for immunoprecipitation. Protein immunoprecipitation was done for 3 h at 4 °C. Later, beads were washed five times in lysis buffer, resuspended in Laemmli sample buffer and boiled for 5 min. Proteins from cell lysates and immunoprecipitation (IP) samples were resolved by SDS-PAGE and blotted according to the above-described protocol.

### Epitope mapping

Peptide synthesis and epitope mapping of the anti-CD95 monoclonal antibodies 9C11 (IgG2b) and 4F10 (IgG2b) was performed by Creative Biolabs (Shirley, NY, USA). 20 overlapping peptides were synthesized and biotinylated with >95% purity. Then, the binding activity of 9C11 and 4F10 was carried out for all peptides. Briefly, antibodies were coated (500 ng/well), washed and non-specific binding sites were blocked with 3% milk. Biotinylated-peptide were added (50 μM), washed and revealed by streptavidin-HRP in the presence of its substrate, Tetramethylbenzidine (TMB). The measured O.D. value obtained with full-length peptide (P1) served as the maximum signal.

### Surface Plasmon resonance experiments

Experiments were performed at 25 °C with a Biacore^™^ T200 apparatus (Biacore^™^, GE Healthcare Life Sciences, Uppsala, Sweden). The experiments were performed on CM5 sensor chips (Biacore^™^) coated with 140 resonance units (RU) of FasL (Peprotech). A flow cell left blank was used for double-referencing of the sensorgrams. Soluble full-length extracellular domain of CD95 (sCD95) or Δ58 truncated constructs (sCD95-Δ58) were prepared in the running buffer, 10 mM Na_2_HPO_4_, 150 mM NaCl, 3 mM EDTA and 0.05% Tween-20 at pH 7.4 and injected in triplicate at 25 µl/min. The regeneration of the functionalized surface was achieved with a 1-min pulse of 0.5% SDS. The sensorgrams were processed using Biacore T200 Evaluation Software 2.0 (Biacore^™^). The association and dissociation rate constants, k_a_ and k_d_, were determined by direct curve fitting of the sensorgrams to a Langmuir 1:1 model of interaction. The dissociation equilibrium constant, K_D_, was calculated as k_d_/k_a_.

### Flow cytometry

CD95 surface expression was determined by cell staining with anti-CD95 or isotype control antibodies. Briefly, cells (2.10^5^) were washed in PBS and blocked for 30 min with PBS containing 1% bovine serum albumin (BSA). Cells were then incubated for 20 min on ice with an anti-CD95 or isotype antibody in PBS-BSA buffer. Then, cells were washed in PBS supplemented with 2% BSA and resuspended in 150 μL of same solution. Staining was analysed immediately by flow cytometry using a BD FACSCalibur^TM^ and the Cell Quest software (BD Bioscience, San Jose, USA). To decrease antibody internalization and background all steps were performed at 4 °C.

### Confocal fluorescence microscopy

Cells were fixed with 4% PFA and stained with the indicated antibodies. Fluorescence microphotographs were taken using a laser scanning confocal system (TCS SP8 model mounted on a DMI 6000 CS inverted microscope, Leica, Heidelberg, Germany). Images were taken using a 63x oil-immersion objective (1.4 N.A.), collected in a format of 1,024 ×1,024 pixels and analyzed using the ImageJ software (NIH, Bethesda, Maryland, USA).

### Proximity ligation assay (Duolink)

Proximity ligation assays (PLAs) were performed on fixed cells in 96 multiwells plates. PLA was carried out using the DUOLink^TM^ kit (OLINK, Uppsala Sweeden) according to the manufacturer’s instructions. After blocking, antibodies were used at the following concentrations: anti-CD95 (DX2) 1:100 and anti-CD95 (N18) 1:100. The number of in situ PLA signals per cell was counted by using the ImageJ software (NIH, Bethesda, Maryland, USA).

### Generation of PLAD-targeting hybridoma

Monoclonal antibodies targeting the amino-terminal region of CD95 were generated with Biotem (Apprieu, France). Briefly, three peptides were synthesized (Peptide1- NSKGLELRKTVTTVETQNLEGC, the last C was added to conjugate the peptide with KLH, Peptide2- ETQNLEGLHHDGQFC and Peptide3- GQFCHKPCPPGERKARDC), conjugated with KLH (keyhole limpet hemocyanin) prior injection in Balb/c mice. Five mice (Oncins France 1 strain) were injected three times at three-week intervals with the KLH-conjugated peptides. The first injection was made subcutaneously and intraperitonealy with complete Freund’s adjuvant and the two others intraperitonealy with incomplete Freund’s adjuvant. Ten days after the third immunization, the levels of serum immunoglobulins were tested by indirect ELISA against the peptides conjugated to BSA by ELISA. Three weeks after the last injection, a selected mouse was given an intravenous injection of peptides conjugated to KLH without adjuvant. Three days after the boost injection, the mouse was sacrificed, and the spleen was taken for lymphocytes isolation. The lymphocytes were fused with Sp2/0-Ag14 myeloma cell line using standard hybridoma preparation. The hybridoma supernatants from the fusion were screened for the presence of antibodies reacting with peptides conjugated to BSA by ELISA. The positive hybridoma were confirmed using flow cytometry to select the best antibodies recognizing the amino-terminal region of CD95 (full length) but not the Δ58 construct.

### Cell proliferation assay

Proliferation was assayed by cell counting. Briefly, cells were cultured in 6-well dishes in duplicate at a density of 5,000 or 20,000 cells as indicated per well in 2 ml of medium supplemented with or without FBS. Medium was changed every 48 h. Cell number at the indicated time points was determined by counting using a haemocytometer.

### CD95 ELISA

Gel filtration fractions were harvested (1 mL) and soluble CD95 constructs were dosed by ELISA following the manufacturer’s instructions (Abcam).

### Computer modeling

The building of a complete CD95 extracellular protomer started from the most complete crystal structure of the receptor (D55 to E167, PDB:3TJE [14]). The lacking Ct loop (connecting to the TM) in the crystal structure (K148 to E156) was built ab initio using Prime, with a VSGB solvation model and OPLS3e force field, as implemented in Maestro 12.4 (Schrödinger LLC, NY). The lacking Nt part (R17 to Q47) was built by a threading approach, as described previously [15]. After splicing of each additional fragment to the crystal structure, the completed protomer was embedded in a solvent box of water molecules and Cl^-^ ions for periodic boundary conditions. 16376 H_2_O and 4 Cl^−^ were used for the wild type protomer, 16683 H_2_O and 3 Cl^-^ for the K33G mutant, 16807 H_2_O and 2 Cl^−^ for the Δ36- truncated protomer. The system was optimized through energy minimization with Desmond implemented in Maestro. Then the system was equilibrated for 60 ns by molecular dynamics, with amino acids present in the crystal structure kept frozen. NPγT conditions were defined with a Nose-Hoover thermostat and Martyna-Tobias-Klein barostat, T = 300 K and P = 1 bar. A multiple time step scheme was used with RESPA integrator (2 fs for bounded and close atoms, 6 fs for the far region). Coulombic interactions were calculated with a 9 Å cutoff. Finally, a production experiment of 2.1 µs was run, without any constraint. Clustering of the trajectory was performed to select the most populated states of the protomer. The corresponding conformer was thereafter submitted to protein-protein docking with SymmDock [16], forcing a dimeric or trimeric arrangement as previously described [17]. The interaction energy of the oligomers was estimated *via* molecular mechanics energies combined with the generalized Born and surface area continuum solvation (Prime-MM/GBSA) approach, using VSGB solvation model and OPLSe force field, and after energy minimization of the system.

### Mass spectrometry

Plasma membrane CD95 was immunoprecipitated from CEM or H9 cell with the anti-CD95 mAb APO1-3. After several washes to clean unbounded antibodies, cells were lyzed for 30 min in a HEPES-Triton X-100 based lysis buffer on ice. CD95 immunoprecipitation was resolved by a 10% SDS gel and coomassie blue staining was used for protein identification. A band around 40-50 kDa corresponding to CD95 present in parental CEM and H9 cells but absent in CD95-KO counterparts was cut. Mass spectrometry and data analysis were done by Alphalyse as described by the company. Briefly, protein samples were reduced, alkylated, and subsequently digested with trypsin. Resulting peptides were concentrated by Speed Vac lyophilization and re-dissolved for injection on a Dionex nano-LC system and MS/MS analysis on a Bruker Maxis Impact QTOF instrument. The MS/MS spectra were used for Mascot database searching. The data are searched against in-house protein databases downloaded from UniProt and NCBI.

### Statistical analysis

All data are displayed as mean values from at least 3 independent experiments. Statistical differences among cell lines or treatments were done by ANOVA test for multiple comparisons, or by paired Student *t* test, as appropriate using the Prism 6.0 (graphPad software). Values with a P value < 0.05 were considered statistically significant.

## Results

### The CD95 N-terminal region is lost in cancer cells and in most of normal cells

The PLADs of TNFR1 and CD95 are involved in receptor aggregation, an essential molecular event for cell signaling [10, 18] principally the induction of the apoptotic signal [19]. Although TNFR1 and CD95 are naturally expressed in many tissues and cell lines, it proved difficult to generate stable cell lines that overexpress these full-length receptors, probably because overexpression might promote their aggregation and the subsequent activation of the apoptotic machinery. In agreement with this hypothesis, silencer of death domains (SODD) prevents the spontaneous induction of cell death by TNFR1 or DR3 [20]. However, such negative regulatory protein has never been identified for CD95, suggesting that an alternative mechanism might be involved to prevent any deleterious ligand-independent aggregation of this particular receptor.

**Fig. 1:**
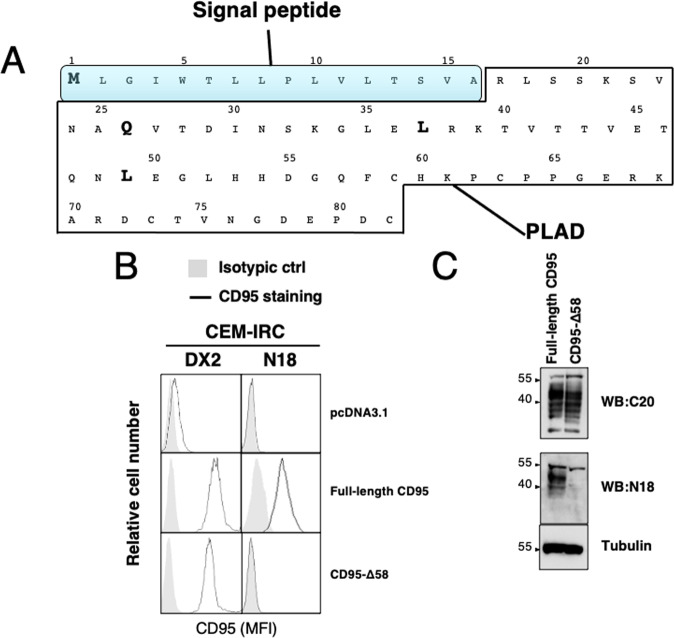
Loss of the N-terminal region of the CD95 receptor. **A** Schematic representation of the N-terminal region of CD95 with the signal peptide depicted in blue. The PLAD is indicated. Bold amino acids represent the first putative amino acids of mature CD95 proteins identified using terminal amine isotopic labeling of substrates (TAILS; http://clipserve.clip.ubc.ca/topfind/). **B** CD95-deficient CEM-IRC cells were transfected with the indicated cDNA-encoding vectors. Clones expressing similar expression level of the indicated CD95 constructs were selected regarding DX2 staining. Plasma membrane expression of CD95 was evaluated by flow cytometry after incubation with the anti-CD95 DX2 or N18 antibodies (open histograms) or with isotype-matched control IgG (shadowed histograms). **C** Immunoblot analyses of total cell lysates from full-length CD95 or CD95-Δ58 transfected CEM-IRC cells. CD95 expression was revealed using C20 an antibody targeting the C-terminal region of CD95 or N18, which recognizes the N-terminal CD95 region. Tubulin serves as a loading control.

CD95 cloning and characterization initially identified a signal peptide (SP) covering amino acids 1 to 16 suggesting that the first amino acid in the mature protein was arginine 17 [21]. Yet, the terminal amine isotopic labeling of substrates (TAILs) method (TopFIND-http://clipserve.clip.ubc.ca/topfind) [22] points out four different N-terminal regions for CD95 including receptors starting at position 1 (methionine), 26 (glutamine), 37 (leucine) or 49 (leucine) (Fig. 1A). This raises the questions of the exact start(s) of the mature CD95 proteins and of the relative abondance and biological significance of these N-terminally truncated CD95 isoforms. Indeed, the PLAD was initially reported to consist in the polypeptide sequence from the arginine at position 17 to the cysteine at position 82 [10], but we further refined the mapping of this region to amino acid residues 59 to 82 [11]. Because the PLAD is starting after any of the TAILs predicted cleavage sites, the biological role of the CD95 amino terminal domain preceding it (residues 17 to 58) remains to be elucidated. To address this question, we have reconstituted a CD95-deficient cell line, namely CEM-IRC [23], with full-length CD95 or a N-terminally truncated CD95 receptor (CD95-Δ58) lacking the 42 amino acid residues following the SP (*i.e*., amino acids 17 to 58, see Fig. 1A, B). Next, we compared the staining pattern of three different anti-CD95 antibodies including N18, a polyclonal antibody recognizing the N-terminal region (from amino acid residues 21 to 38) [24, 25], DX2, a monoclonal antibody binding to an extracellular epitope localized between CRD2 and CRD3 [14] and the polyclonal antibody C20, targeting the intracellular C-terminal region (amino acids 316-335) (Schmitz et al., 2002) (Supporting Fig. 1A). The expression level of the CD95 constructs at the plasma membrane was evaluated by flow cytometry (Fig. 1B). The whole quantity of CD95 was analyzed by immunoblotting (Fig. 1C & supplementary raw data). While N18 recognized full-length CD95, it failed to bind the truncated receptor confirming its selectivity for the extreme amino-terminal region of CD95 (Fig. 1B, C). As expected, C20 and DX2 recognized both full-length and CD95-Δ58 (Fig. 1B, C). Confocal microscopy (Fig. S1B) and proximity ligation assay (PLA) (Fig. S1C) showed that DX2 and N18 staining co-localized in full-length CD95-expressing cells whereas only DX2 stained CD95-Δ58 cells (Fig. S1B). Overall, these findings confirmed that the N18 antibody recognized the N-term region of CD95, upstream of the cysteine at position 59.

**Fig. 2:**
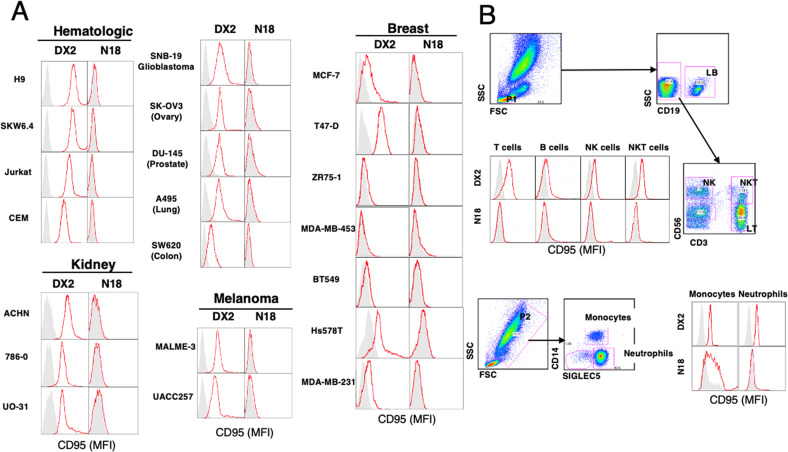
The amino-terminal region of CD95 is lost in all cancer cells and normal blood cells but macrophages. **A** Indicated cancer cell lines were stained with DX2 and N18 as primary antibodies (red lines) or with isotype-matched control IgG (shadowed histogram) and CD95 expression was evaluated by flow cytometry. **B** Normal human peripheral blood cells (PBLs) were stained by DX2 or N18 (red lines) or isotype-matched control IgG (shadowed histogram) and CD95 expression was analyzed by flow cytometry. Cell populations were evaluated by using specific markers (T cells (CD3^+^), B cells (CD19^+^); NK cells (CD56^+^), NKT cells (CD3^+^CD56^+^), monocytes (CD14^+^SIGLEC5^+^) and neutrophils (CD14^−^SIGLEC5^+^)). Note that the N-terminal domain of CD95 was only detected in the monocyte population.

Interestingly, although all cancer cell lines except MDA-MB-453 (i.e., 21 cancer cell lines derived from various histological origins) expressed CD95 at their plasma membrane as revealed with the DX2 staining (Fig. 2A), N18 did not show any staining (Fig. 2A). To determine whether this observation was specific to transformed cells, similar experiments were performed on blood cells from healthy donors. This revealed that most immune cells such as T- (CD3^+^CD56^−^) and B- (CD19^+^) lymphocytes, NK cells (CD3^−^CD56^+^), NKT cells (CD3^+^CD56^+^) and neutrophils (CD14^−^SIGLEC5^+^) had also lost their N18 epitope even though these cells expressed CD95 as revealed by DX2 staining (Fig. 2B). Among all tested cells, only monocytes (CD14^+^SIGLEC5^+^) showed a heterogeneous but positive staining for N18 (Fig. 2B).

### Endogenous CD95 lacks its most N-terminal domain

Two possible explanations for the inability of the N18 antibody to detect endogenous CD95, are either that post-translational modifications (PTM) such as steric hindrance of sugar chains, interfere with its binding, or that the N18 epitope is simply lost in endogenous CD95. Regarding PTM, despite N118 and N136 were shown as the main N-glycosylation residues in CD95 [26], an in silico analysis of the CD95 N-term region covering amino acid residues 17 to 70 predicted one putative additional N-glycosylation site at asparagine in position 31 and four O-glycosylation sites at serines 19, 20 and threonines 40 and 46 (Fig. S2A) [27–29]. To address a possible interference of CD95 glycosylation with N18 binding, the soluble extracellular region of CD95 (sCD95) consisting in amino acid residues 1 to 174 (Fig. S2B) was produced as secreted and fully glycosylated protein in the eukaryotic HEK/293 T cells (Fig. S2C & supplementary raw data). Immunoblotting analyses further revealed that sCD95 is perfectly detected by the N18 antibody arguing against a possible glycosylation-mediated interference with N18 binding. To further confirmed this hypothesis, sCD95 glycosylation were removed by enzymatic treatments. While N-glycosidase caused a shift of sCD95 migration in SDS-PAGE, O-glycosidase did not (Fig. S2C) pointing out that the extracellular region of CD95 underwent N-glycosylation only. Because the elimination of CD95 N-glycosylation did not boost the N18 staining (Fig. S2C), we concluded that this type of PTM was not involved in the observed loss of N18 staining. Therefore, we wondered if the N18 epitope could be lost due to mRNA splicing. While many isoforms of CD95 exist, no splice variants of exon 2 coding for the CRD1 domain have ever been reported [1, 10, 25]. In addition, although the N18 staining was lost at the plasma membrane (Fig. 2A and S2D), N18 was still able to detect intracellular CD95 receptors after cell permeabilization (Fig. S2D) as demonstrated by co-localization with DX2 staining (Fig. S2D). These findings indicated that the loss of the N18 epitope occurred post-translationally and ruled out alternative CD95 splicing to explain this phenomenon.

### MMPs are involved in the cleavage of the CD95 amino terminal domain

**Fig. 3:**
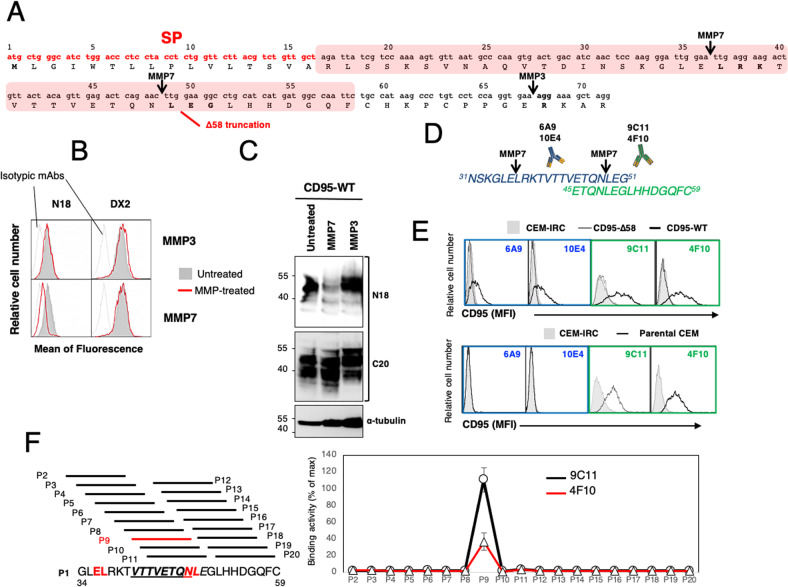
MMP7-mediated cleavage of the CD95 N-terminal domain. **A** Schematic representation of the predicted metalloprotease (MMP) cleavage sites in the amino terminal region of CD95 [26]. Box in red refers to the truncated CD95-Δ58 construct. SP refers to signal peptide. **B** CEM-IRC cells reconstituted with full-length CD95 were incubated with (red line) or without (filled grey curve) recombinant MMP-3 or MMP-7 (0.1 µg/ml) for 4 h at 37 °C and CD95 expression was assessed by flow cytometry using N18 or DX2. Isotypic antibodies for N18 and DX2 are depicted. **C** CEM-IRC cells reconstituted with full-length CD95 (CD95-WT) were treated as in (B), lyzed and indicated western blotting were performed. Tubulin immunoblot serves as loading control. **D** The two peptides covering the N-terminal region of CD95 have been injected in mice to generate anti-CD95 mAbs. **E** Flow cytometry analyses of the CD95 constructs recognized by monoclonal antibodies in (**D**). 9C11 and 4F10 recognize endogenous CD95 (parental cells), while 6A9 and 10E4 fails to recognize it. (**F**). Epitope mapping of 9C11 and 4F10 antibodies as described in Materials and methods. Left panel; indicated overlapping peptides covering the CD95 amino acid residues 34 to 59 are used for epitope mapping. Red amino acids represent the two putative cleavage sites of MMP7. The underlined sequence corresponds to the epitope recognized by 9C11 and 4F10. *Right panel*; histograms represent ELISA values for each indicated peptide for 9C11 or 4F10 (ratio of the value for each peptide to the maximum value corresponding to full-length sequence signal).

It has been shown that MMP7 can cleave CD95 in its CRD1 between amino acids ^36^EL^37^ and ^48^NL^49^ leading to the down-regulation of its apoptotic signaling pathway [30]. Hence, we have used the PROSPER server and search for in silico prediction of protease cleavage sites [31]. This revealed that not only MMP7 but also MMP3 could potentially cleave the extracellular region of CD95 (Fig. 3A). To determine whether MMP3 and/or MMP7-dependent CD95 processing were responsible for the loss of the N18 staining through epitope removal, we incubated full-length CD95-expressing CEM-IRC cells with recombinant MMP3 or MMP7 and evaluated the ability of N18 and DX2 antibodies to detect CD95 by flow cytometry (Fig. 3B). While recombinant MMP7 reduced N18 staining in CD95-expressing T-cells (Fig. 3B), MMP3 treatment did not (Fig. 3B). Similar data were obtained by analyzing this staining by western blot confirming that only MMP7 was able to cleave the amino-terminal region of CD95 encompassing the N18 epitope (Fig. 3C & supplementary raw data). To next identify where the CD95 was cleaved within its amino terminal extremity, we immunoprecipitated plasma membrane CD95 endogenously expressed in leukemic T-cells and sequenced the amino terminal region. To do so, cells were pre-incubated with the agonist anti-CD95 mAb APO1-3, washed to eliminate the excess of antibody and then lyzed before performing the immunoprecipitation. Membrane CD95 was next analyzed through LC–MS/MS. Using LC–MS/MS, we found that the first peptide corresponded to residues 71–100 (RDCTVNGDEPDCVPCQEGKEYTDKAHFSSK) (Fig. S2E), suggesting that either endogenous CD95 lost its first 70 amino acid residues or that this proteomic approach failed to detect the presence of a more amino terminal amino acid region probably due to post-translational modifications of the protein (*i.e*., formylation, acetylation) or disulfide bridge between two cysteines sequestering the N-terminus. To better map the region of CD95 cleaved by MMP7, peptides covering the amino acid residues 31–59 that encompass the two putative MMP7 cleavage sites (i.e., ^36^EL^37^ and ^48^NL^49^) were used to generate monoclonal antibodies (Fig. 3D). Among the selected hybridoma, the 6A9 and 10E4 mAbs were obtained for mice immunized with the ^31^NSKGLELRKTVTTVETQNLEG^51^ peptide, while the 9C11 and 4F10 were obtained using the ^45^ETQNLEGLHHDGQFC^59^ sequence (Fig. 3D). Although all mAbs recognized full-length CD95 when overexpressed in CEM-IRC cells (Fig. 3E), none of them interacted with the CD95-Δ58 constructs (Fig. 3E) confirming that the generated antibodies bound epitopes upstream the cysteine at position 59. Interestingly, whereas 9C11 and 4F10 mAbs stained endogenous CD95 in parental CEM T-cells (Fig. 3E), 6A9 and 10E4 mAbs failed to do so (Fig. 3E) supporting that endogenous CD95 still possessed the ^45^ETQNLEGLHHDGQFC^59^ sequence, which includes the putative MMP7 cleavage site ^48^NL^49^. To narrow this region down, we conducted an epitope mapping of 9C11 and 4F10 using CD95 amino acid residues 34 to 59 (Fig. 3F). This showed that 9C11 and 4F10 recognized the same epitope, namely ^41^VTTVETQN^48^ (Fig. 3F) and ruled out the implication of ^48^NL^49^ as the main CD95 cleavage site while strongly supporting that the receptor was processed between glutamic acid 36 and leucine 37 by MMP7.

Surprisingly, although the CD95 N-terminal-recognizing N18 (see Fig. 2A), 6A9 and 10E4 (Fig. 3E) antibodies failed to detect endogenous CD95 in parental CEM cells, they still stained the full-length CD95 when overexpressed in CEM-IRC cells (see Figs. 1B, E). A possible explanation for this discrepancy is that the CMV promoter-driven robust and constitutive expression of full-length CD95 in CEM-IRC cells could overwhelm the endogenous MMP7-mediated cleavage rate and by doing so, allowed a certain proportion of full-length receptor to escape from MMP7 cleavage. In agreement with this leakage hypothesis, we noticed a lower intensity of the N18 staining in CEM-IRC cells expressing full-length CD95 as compared to the one obtained with DX2 (Fig. 1B and Fig. 3B) and similarly, only a reduced quantity of full-length CD95 can be detected with 6A9 and 10E4 antibodies as compared to those obtained with 9C11 and 4F10 mAbs (Fig. 3E).

### N-terminal CD95 cleavage reduces the magnitude of CD95 aggregation

**Fig. 4:**
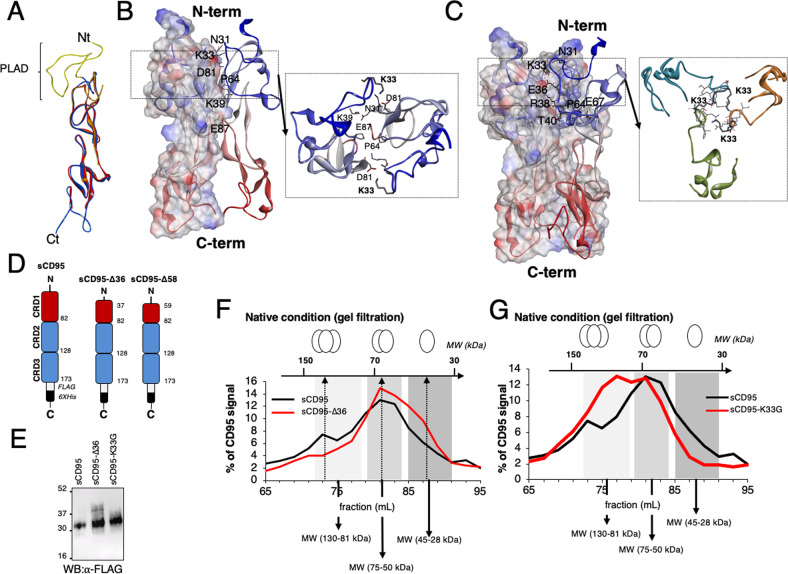
The N-terminal region of CD95 contributes to receptor aggregation. **A** Superimposition of the two incomplete crystal structures (PDB:3THM in orange and PDB:3TJE in red). Using these structures, the complete amino-terminal region from R17 to G51 in PLAD was reconstituted by ab initio modeling and fused to the X-ray CD95 structures (in blue). Long molecular dynamics experiments (2µs) were finally performed to equilibrate the model. **B** The 3D organization of the CD95 dimeric model was generated by protein-protein docking of the protomer using SymmDock, with an imposed 2-fold symmetry. Most important amino acid involved in the interaction are depicted. **C** The 3D organization of the CD95 trimeric model was generated by protein-protein docking of the protomer using SymmDock, with an imposed 3-fold symmetry. **D** Indicated soluble CD95 constructs were expressed in HEK/293 T cells. **E** In denaturing and reducing conditions, the molecular weights of the soluble forms of full-length extracellular domain of CD95 (sCD95), Δ36 truncated counterpart (sCD95-Δ36) and K33G mutant (sCD95-K33G) were assessed by western blot. **F** In native condition, supernatants of the different sCD95-secreting HEK/293 T cells were resolved by gel filtration and, the quantity of sCD95 present in each fraction was measured by ELISA. The molecular weights of full-length extracellular domain of CD95 (sCD95) and sCD95-Δ36 constructs were assessed using native molecular markers. For each fraction, the percentage of dosed CD95 was depicted. **G** In native condition, the molecular weights of full-length extracellular domain of CD95 (sCD95) (also depicted in **F**) and sCD95-K33G constructs were assessed by gel filtration as described in (**F**).

To gain insight on the possible impact of MMP7 cleavage on CD95 function, we first performed a structural approach. Although two different X-ray structures of CD95 exist, its amino terminal region has not been yet elucidated: the solved amino terminal sequence starts with aspartic acid at position 55 (PDB:3TJE) or leucine at position 52 (PDB:3THM) [14]. The lack of electron density for the most amino terminal region of CD95 (residues 17 to 58) suggests that it is a flexible and disordered domain. This was confirmed by molecular dynamics experiments (Fig. S3A–B) with a mean RMSD of 6.6 Å for residues R17-G56 during the simulation, as compared to 4.7 Å for Q57-T147. Therefore, we reconstituted the full-length CD95 protein by combining available X-ray data and computer modeling (Fig. 4A).

Biochemical and imaging data [10, 18] indicated that TNFR members homotrimerize in a ligand-independent fashion, while other studies proposed that these receptors are present at the plasma membrane as pre-associated homodimers based on X-ray analyses [32] and single-molecule imaging [33]. Hence, because no consensus exists on TNFR stoichiometry in the absence of the ligand, we used 2-fold (Figs. 4B) and 3-fold (Fig. 4C) symmetries to analyze the behavior of the CD95 N-terminal region in the context of both homodimer and homotrimer. These computer-driven models suggested that dimeric and trimeric interfaces were not exactly the same, even if some residues were conserved, including N31, K33 and P64. The dimer interface possessed few specific side-chain interactions and the most important ones corresponded to N31/K33 of one protomer with D81 of the other, K39 with E87 and P64 with P64 (Fig. 4B, C). The trimer interface involved more amino acids, among which N31, E36, R38, K39, T40, P64, P65 and E67. In agreement with the presence of many interacting residues in the amino-terminal extremity of trimeric CD95, the full-length extracellular region of CD95 displayed a higher interaction energy than its MMP7 truncated Δ36 counterpart (Table 1). To confirm these calculations, we generated experimentally three CD95 proteins consisting of the full-length extracellular region of CD95 (soluble CD95 or sCD95, Fig. S2B) and the truncated Δ58 and Δ36 counterparts (corresponding respectively to the pre-PLAD sequence [11] and the MMP7-cleaved form, Fig. 4D). These secreted constructs were produced in HEK/293 T cells (Fig. 4D, E and S3C & supplementary raw data) and their stoichiometry was evaluated in native conditions using size exclusion chromatography (Fig. 4F–G and S3D & supplementary raw data). Although in reducing and denaturing conditions, soluble forms of full-length and Δ36-CD95 migrated at close molecular weights (~30 kDa) (Fig. 4E & supplementary raw data), in native condition and after exclusion chromatography experiments, the sCD95 (44%) and sCD95-Δ36 (50%) constructs were resolved between fractions 79 and 85 (mL) corresponding to 75 and 45 kDa, respectively (Fig. 4F). These data indicated that the extracellular region of CD95 predominantly formed dimeric complexes (Fig. 4F). Interestingly, the percentage of monomer that are resolved between fractions 85 and 91 (corresponding to the 45-29 kDa MW range) was increased in sCD95-Δ36 (30%) as compared to CD95 (20%), while the percentage of homotrimer dropped (Fig. 4F; 19% of trimer for sCD95-Δ36 and 27% for sCD95), suggesting that the loss of the first 36 amino acids of CD95 impinged on the formation of CD95 homotrimers at the benefit of monomers (Fig. 4F). In agreement with these data, deleting the first 58 amino acids as initially reported in the PLAD studies [10, 25], also engendered a shift toward lower molecular weights monomers as compared to CD95 (Fig. S3D).

**Table 1:** CD95 trimer interaction energy.

	trimer		
	WT	Δ36	K33G
E_int (kcal/mol)_	−89.9	−72.2	−97.2

Calculated interaction energy of the different constructs of CD95 oligomers.

According to our in silico modeling, although CD95 K33 contributed favorably to dimer interaction (Fig. 4B), in the trimeric model face to face positioning of K33 in each protomer forces its alkyl chain to bend in order to avoid electrostatic clash (Fig. 4C). Hence, our computer-driven model suggested a detrimental effect of K33 in the formation/stability of trimeric complex. To evaluate this model, we generated a soluble K33G CD95 mutant (sCD95 K33G) and expressed it in HEK/293 T cells (Fig. 4E) to compare its stoichiometry to its wild type counterpart using size exclusion chromatography (Fig. 4G). In agreement with the computer-driven model, we observed that replacement of K33 by a glycine (i.e., a flexible amino acid with no side chain) favored the formation of a trimeric structure as compared to the wild type receptor (Fig. 4G). Molecular modeling analyses of the trimeric wild type, CD95-Δ36 and CD95-K33G constructs confirmed that the interaction energy was stronger for K33G mutant as compared to wild type and CD95-Δ36 constructs (Table 1). The lower flexibility of the protomer bearing K33G as compared to its wild type counterpart also explained its higher propensity to form trimers (Fig. S3B and Table 2). Overall, these findings pointed out that metalloprotease-cleavage of the amino-terminal region of CD95 fine-tunes the magnitude of CD95 aggregation through structural reorganization.

**Table 2:** Stability of K33G CD95 mutant.

	Nt			RX	
	wt	K33G		wt	K33G
RMSD (Å)	6.6	5		4.7	2.8

Mean RMSD of rebuilt (Nt) and crystal structure (RX) of CD95 during 2 µs molecular dynamics.

### Intact CD95 N-terminus enhances the magnitude of the apoptotic program

**Fig. 5:**
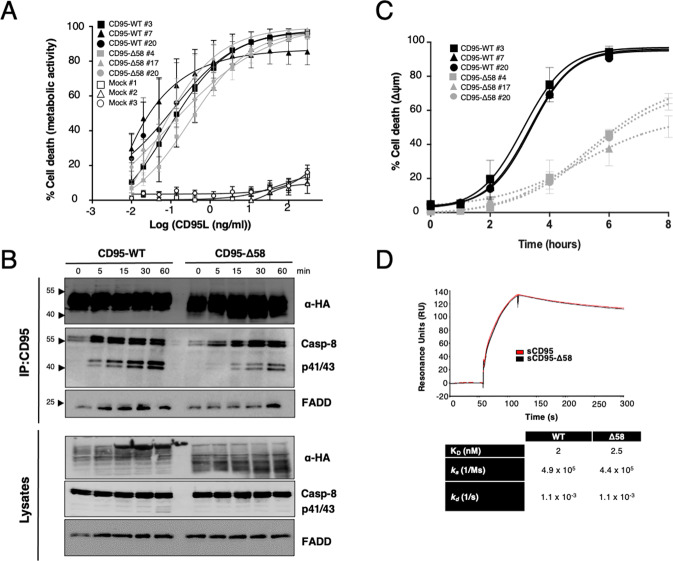
Elimination of the CD95 amino terminal region impinges on the apoptotic signal. **A** CEM-IRC clones stably expressing full-length CD95, CD95-Δ58 or empty vectors were incubated for 24 h with the indicated concentrations of cytotoxic IgCD95L and cell death was assessed by MTT assay. Data represent mean ± SD of three independent experiments. **B** Full-length or CD95-Δ58-expressing CEM-IRC clones were incubated with IgCD95L (100 ng/mL) for the indicated times. Cells were then lyzed and CD95 was immunoprecipitated using the APO1-3 antibody. The quantity of co-immunoprecipitated FADD and caspase-8 was evaluated by immunoblotting. Data are representative of three independent experiments. **C** Full-length and CD95-Δ58-expressing CEM-IRC cells were incubated for indicated time points with IgCD95L (100 ng/ml) and the drop of mitochondrial potential (ΔΨ_*m*_) was assessed. Data represent mean ± SD of three independent experiments. **D** Surface plasmon resonance analyses of CD95L binding to immobilized soluble full-length extracellular domain of CD95 (sCD95) or its Δ58 counterpart (sCD95-Δ58). The kinetic parameters are listed in the bottom table.

Next, we wondered whether and how the loss of the CD95 amino terminal extremity could affect the induction of the apoptotic signaling pathway. Because Δ58 and Δ36 constructs exhibited similar aggregation patterns and initial studies on the PLAD were carried out using CD95-Δ58 constructs [10, 34, 35], we further investigated the CD95 signaling pathways in the CD95-deficient CEM-IRC cells [23] reconstituted with full-length or CD95-Δ58 receptors that we had previously established (Fig. 1). Exposure of these cells to a multi-aggregated and CD95L, namely Ig-CD95L, for 24 h showed that full-length and CD95-Δ58-expressing CEM cells exhibited similar cell death sensitivity (Fig. 5A), while CD95-deficient CEM-IRC cells were indeed resistant to this stimulus. However, the evaluation of the early steps in the CD95-mediated apoptotic processes such as the formation of the DISC (Fig. 5B & supplementary raw data) and the drop of the mitochondrial potential (Fig. 5C) revealed that the progression of the cell death program was slower in CD95-Δ58-expressing cells as compared to full-length CD95-expressing counterparts as illustrated by the delay in the recruitment of the FADD adaptor protein by CD95 and of the downstream activation of the caspase-8 (apparition of the first steps of caspase-8 cleavage with the p41/p43 bands) in CD95-Δ58 cells as compared to wild type CD95 cells (Fig. 5B). To rule out that the elimination of the amino acid residues 1 to 58 in CD95 might have affected receptor/ligand recognition, we assessed the constant affinities (K_D_) of CD95L binding to the full-length extracellular region of CD95 (sCD95) and its counterpart lacking the first 58 amino acids (sCD95-Δ58) using surface plasmon resonance (SPR) (Fig. 5D). The sensorgrams for sCD95 and sCD95-Δ58 were superimposable (Fig. 5D) with estimated K_D_ values (2 nM for sCD95-WT *vs* 2.5 nM for sCD95-Δ58) coherent to those described in CD95-expressing cells [36]. Taken together, these results showed that the N-terminal extremity of CD95 does not contribute to the binding efficiency of CD95L but accelerates the onset of the apoptotic program.

### N-term domain cleavage of CD95 triggers pro-survival signaling pathways

**Fig. 6:**
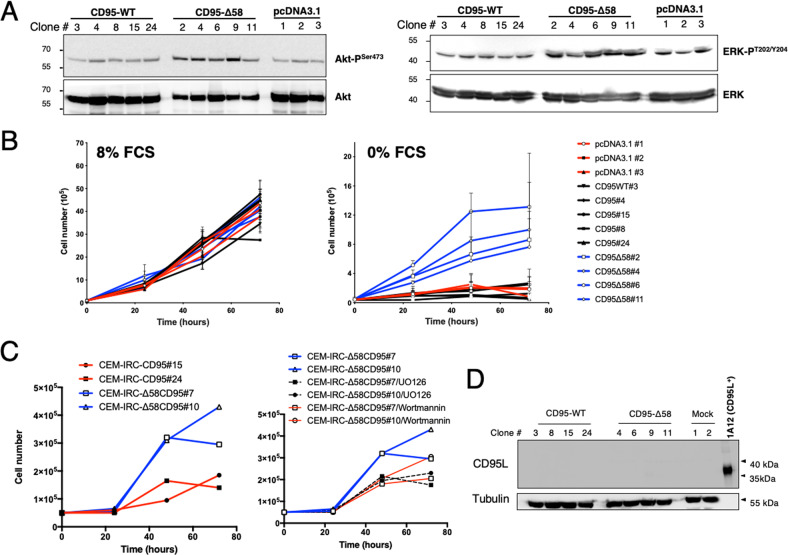
Elimination of the CD95 amino terminal region enhances pro-survival signaling pathways in a CD95L-independent manner. **A** Serum starved CEM-IRC cells reconstituted with full-length CD95, CD95-Δ58 or empty vector were lyzed and the indicated immunoblots were performed. Activation status of the PI3K and MAPK kinase pathways was evaluated by phosphorylation of Akt at Ser473 and ERK at T202/Y204, respectively. Data are representative of three independent experiments. **B** Growth curves for full-length CD95, CD95-Δ58 or empty vector transfected CEM-IRC clones. Cells (10^4^) were plated onto 6-well tissue culture dishes and incubated in RPMI containing 8% or 0% FCS. Cells were counted at 0, 1, 2, and 3 days. **C** Indicated cells were incubated with non-cytotoxic concentrations of the PI3K (wortmannin) and ERK (UO126) inhibitors. Data are representative of three independent experiments. **D** Western blot analysis of total cell lysates from CEM-IRC cells reconstituted with full-length CD95 (CD95), CD95-Δ58 or empty vector (numbering indicates clones). Human CD95L-expressing 1A12 cells serve as positive control. Tubulin is used as a loading control. Note: 1A12 is a mouse T-cell line and the anti-tubulin antibody was specific for human tubulin, therefore mouse tubulin was not recognized in 1A12 cells.

CD95 is a death receptor but, paradoxically, most tumors require a certain level of CD95 expression to be able to survive and proliferate [37]. Several studies including ours have demonstrated that interaction of CD95L with CD95 can induce non-apoptotic signaling pathways promoting tumor progression (i.e., PI3K and ERK) [3, 4, 38] but the molecular mechanism by which CD95 exerts these pro-oncogenic functions remains to be fully elucidated. We therefore investigated whether the induction of these signals could be modulated by the presence of the CD95 amino-terminal region. Interestingly, while CD95-deficient CEM-IRC cells expressing an empty vector or reconstituted with full-length CD95 displayed low levels of phosphorylated Akt and p42/p44, used respectively as hallmarks of PI3K and ERK activations (Fig. 6A & supplementary raw data), both kinases were activated in cells reconstituted with CD95-Δ58 (Fig. 6A). In agreement with the implementation of these pro-survival signaling pathways, only CD95-Δ58-expressing clones survived and proliferated in a serum-deprived medium (Fig. 6B) while no difference was observed in FCS-containing medium. The survival advantage of the CD95-Δ58-expressing clones did rely on the activated PI3K and ERK signaling pathways in these cells, because non-toxic concentrations of a PI3K inhibitor (wortmannin) or a MEK1/2 (i.e., kinases involved in ERK activation) inhibitor (UO126) reduced cell proliferation of CD95-Δ58 clones cultured in serum-free medium (Fig. 6C). Of note, the induction of these pro-survival responses probably occurred in a CD95L-independent fashion because no trace of CD95L expression could be detected in full-length CD95- and Δ58-CEM-IRCs (Fig. 6D & supplementary raw data). Overall, these findings revealed that the elimination of the N-terminal extremity of CD95 might favor pro-survival/proliferative signaling pathways at the expense of the induction of the apoptotic events.

## Discussion

The extracellular amino-terminal region of CD95 encompassing the PLAD is considered to contribute to CD95 homotrimerization [10]. In that region, the amino acid residues 59 to 82 play a major role in CD95L-independent receptor pre-association [11]. In this study, we reveal that the MMP7-shedding of the amino-terminal region of CD95, between glutamic acid at position 36 and leucine at position 37, generates a pro-survival receptor triggering PI3K/MAPK signaling pathways in a CD95L-independent fashion. Although the association between MMPs and TNF ligands has been extensively studied [39] and has shown a shift from the apoptotic function of the membrane-bound ligands to their pro-survival and pro-inflammatory properties, the role of these proteases in death receptor responses remains poorly investigated. This study emphasizes that the MMP7-driven CD95 cleavage modulates the nature (survival versus apoptosis) and intensity (delay of the apoptotic response) of its downstream signaling pathways.

DR5 is one of the tumor-necrosis-factor related apoptosis inducing ligand (TRAIL) receptors. The DR5 transmembrane (TM) helix promotes the assembly of high-order complex promoting the induction of cell death [40]. Interestingly, the elimination of the DR5 extracellular domain triggers cell death in a ligand-independent fashion suggesting that the extracellular region of DR5 exerts an inhibitory effect on receptor activation [40]. Although, the CD95 TM has the tendency to form homotrimers in vitro, mutation in this domain does not prevent ligand-independent receptor pre-association, confirming the pivotal role played by the extracellular domain of CD95 in its stoichiometry and the implementation of the downstream cell signaling [41].

While our data are in agreement with the initial PLAD description [10] showing that both 17–58 (1–42 in the mature protein) and 58–82 (42 to 66 in the mature protein) domains contributed to the homotypic interaction of CD95 [10], we establish that the 17–58 region undergoes a post-translational truncation and that the MMP-driven release of sequence 17-36 modulates the balance between monomer, dimer and trimer of CD95. Patients affected by an autoimmune lymphoproliferative syndrome (ALPS) type 1 A exhibit heterozygous germ line mutations in the CD95 gene [42, 43] and their cells resist to the CD95-mediated apoptotic signal mostly due to mutations accumulated in their death domain [1]. The expression of these CD95 mutations causes dominant interference with the CD95L-induced apoptotic signal. Interestingly, the heterozygous expression of a CD95 mutant deleted of its amino acid residues 68–112 (designated D52-96 (mature protein) or ALPS Pt 2 in [10]) fails to bind CD95L but still inhibits, in a dominant manner, the CD95L-mediated apoptotic signal suggesting that the PLAD is located between the amino acid residues 17 and 67. We can now refine the PLAD to the amino acid residues 37–67. Finally, although the MMP7-driven cleavage of CD95 might account for its pro-oncogenic function [37], its role in normal cells remains to be elucidated, and it also raises the question whether all or a part of the released peptide corresponding to amino acid residues 17 to 36 exerts any biological function.

## Supplementary information

Supporting Figures

Western blot raw data

aj-checklist

## Data Availability

Raw data have been placed in the supporting file “Western blot raw data”. Any additional information required to reanalyze the data reported in this paper is available from the lead contact upon request. Plasmids and cell lines will be provided upon request.
