# Supplementary material for: MMP7 cleavage of amino-terminal CD95 death receptor switches signaling toward non-apoptotic pathways
Source: Cell Death Dis. 2022 Oct 23;13(10):895. doi: 10.1038/s41419-022-05352-0 (PMC9588774; doi:10.1038/s41419-022-05352-0)
Supplement: Supplementary file 1 — Supporting Figures [file 41419_2022_5352_MOESM1_ESM.pdf]

SUPPORTING FIGURES

Supporting figure 1

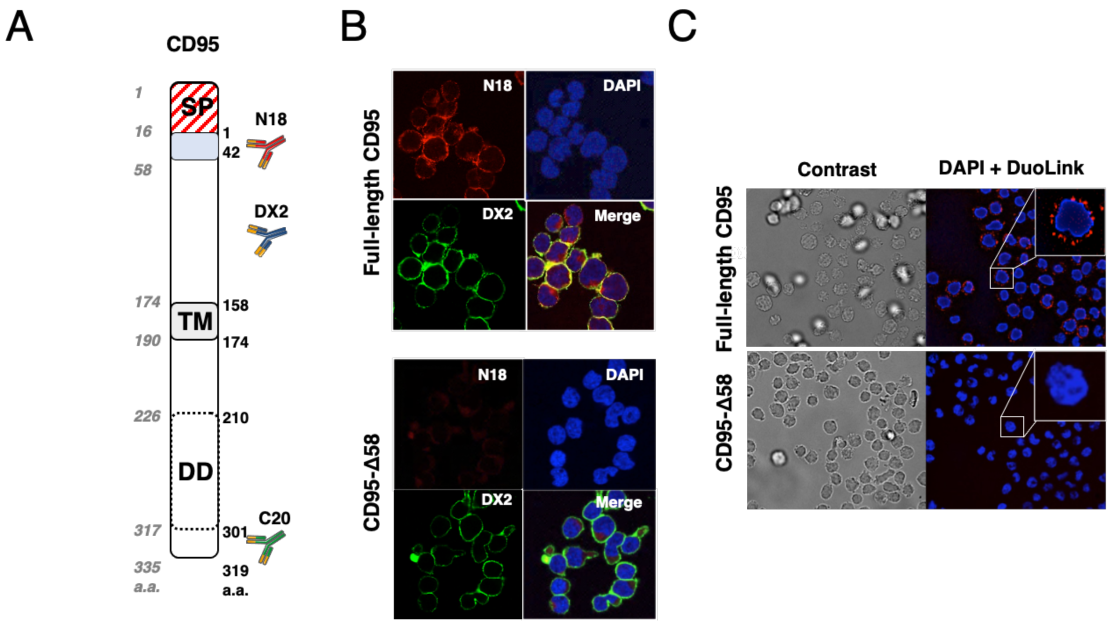

Supporting figure 1. The N18 antibody recognizes a CD95 N-terminal domain preceding the PLAD.

(A). CD95 epitopes recognized by N18, DX2 and C20 antibodies.

(B). Confocal microscopy showing colocalization of anti-CD95 DX2 (green) and N-18 (red) staining in CEM-IRC cells reconstituted with full-length CD95 (upper panels) but not with CD95-Δ58 (lower panels). Nuclei were stained with DAPI (blue).

(C). Confocal analysis of proximity ligation assays (PLAs) showing close proximity (red fluorescent dots) between the DX2 and N18 antibodies in CEM-IRC cells expressing full-length CD95 (top panels) but not in cells expressing the CD95-Δ58 mutant (bottom panels). Box panels show higher magnification images.

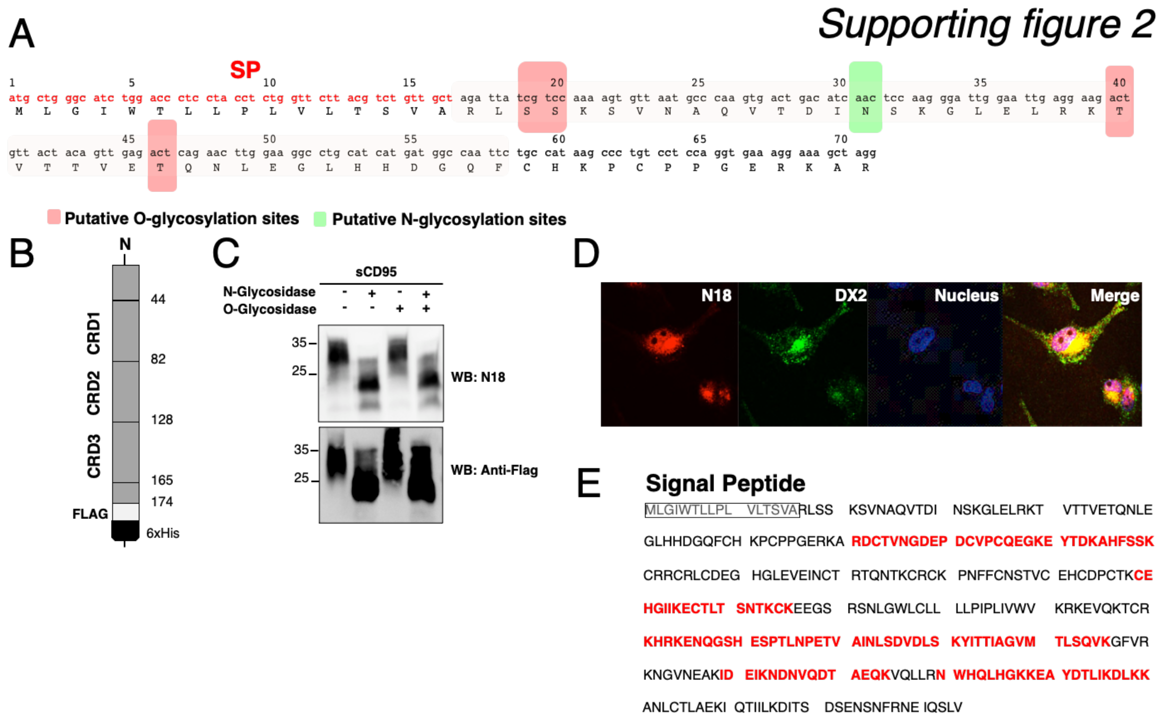

Supporting Figure 2. CD95 glycosylation did not affect N18 binding.

(A). Schematic representation of the predicted glycosylation sites in the first 70 amino acids of CD95. Boxes in red refer to putative O-glycosylation sites and the box in green indicates N-glycosylation site. SP refers to signal peptide.

(B). Schematic representation of the soluble CD95 (sCD95) encoding the extracellular region of CD95 fused to FLAG and 6xHis tags.

(C). Soluble CD95, produced in HEK/293T cells, was treated in the presence or absence of N-glycosidase, O-glycosidase or a combination of both and western blot analyses were performed with the indicated antibodies.

(D). Confocal microscopy analyses of BT549 breast cancer cells stained with anti-CD95 N18 (red) or DX2 (green) antibodies under permeabilized conditions. Nuclei were stained with DAPI (blue).

(E). CD95 was immunoprecipitated in H9 cells and the peptides in red were detected by LC/MS-MS.

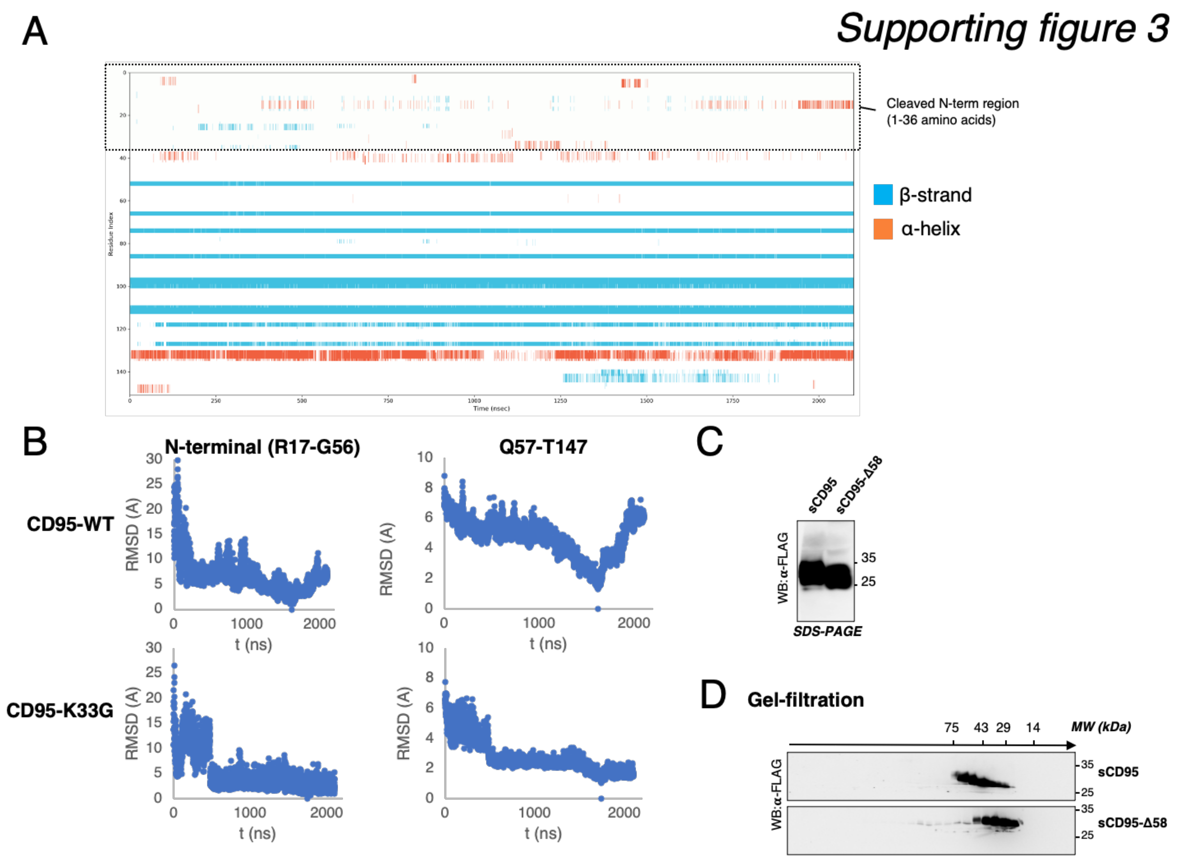

Supporting Figure 3. Structuration of CD95 as determined by molecular dynamics.

(A). Secondary structure composition of the CD95 protomer during molecular dynamics experiment. We remark that unlike the disordered and transitory folded N-terminal region, portion of the protein that was solved by X-ray analysis remains stable (β strands).

(B). Fluctuation of the N-terminal region (R17 to G56), and crystallized portion (Q57 to T147) of the CD95 heavy atoms during molecular dynamics, as measured through RMSD from the most populated conformer cluster (about 1600 ns). Note the high flexibility of the N-terminal part (mean RMSD = 6.6 Å for N terminal part and 4.7 Å for the rest of the wild type protein) as compared to the K33G mutant (mean RMSD = 4.7 Å and 2.8 Å, respectively for the N terminal part and the middle portion).

(C). In denaturing and reducing conditions, the molecular weights of full length, Δ58- and wild type CD95 constructs were assessed by western blot.

(D). In native condition, the molecular weights of full-length and CD95-Δ58 constructs were assessed by western blot with indicated antibody.
