# Supplementary material for: MMP7 cleavage of amino-terminal CD95 death receptor switches signaling toward non-apoptotic pathways
Source: Cell Death Dis. 2022 Oct 23;13(10):895. doi: 10.1038/s41419-022-05352-0 (PMC9588774; doi:10.1038/s41419-022-05352-0)
Supplement: Supplementary file 2 — Western blot raw data [file 41419_2022_5352_MOESM2_ESM.pptx]

## Slide 1
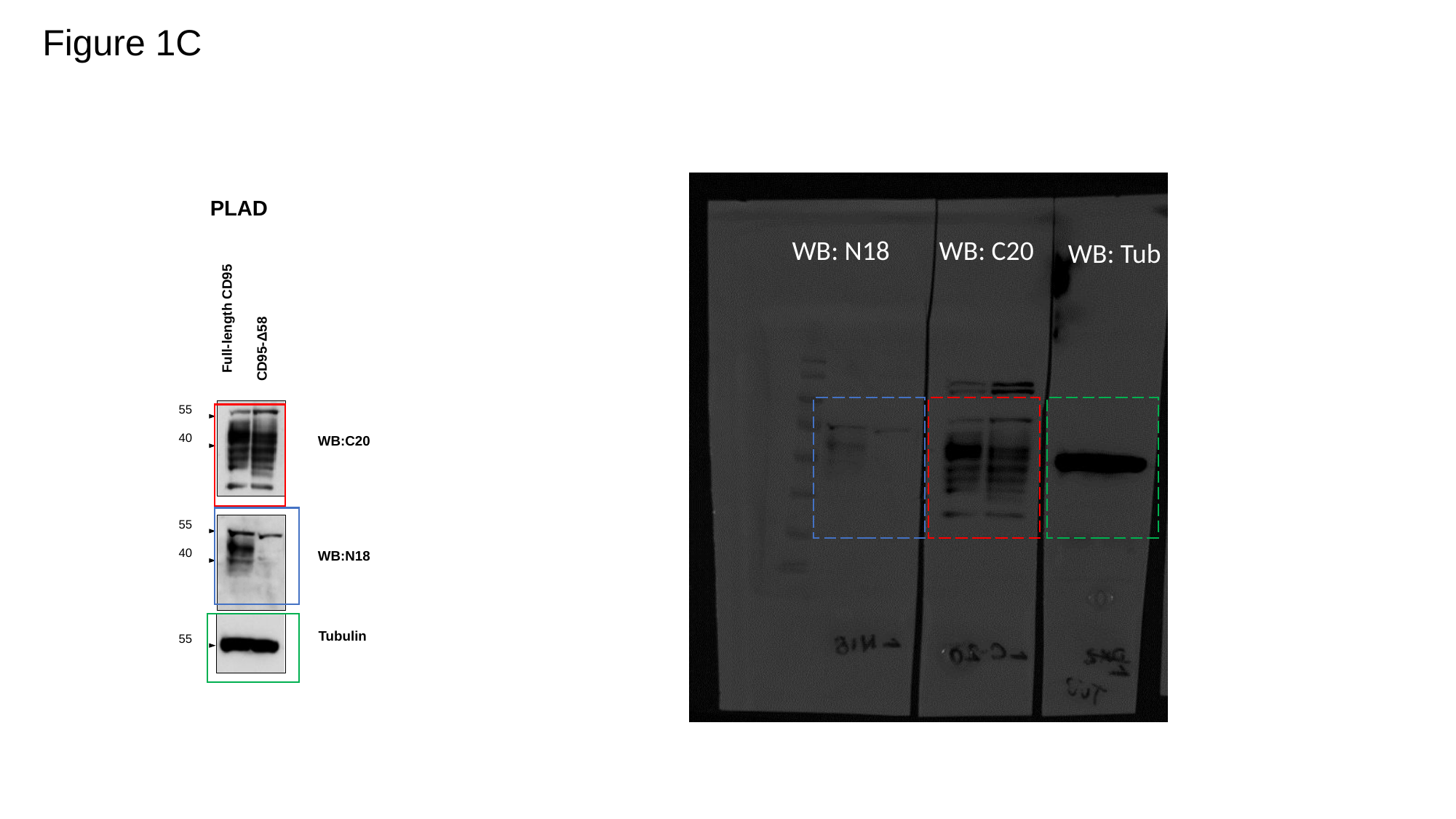

Figure 1C
PLAD
Full-length CD95
CD95-Δ58
55
40
WB:C20
55
40
WB:N18
Tubulin
55
WB: N18
WB: C20
WB: Tub

## Slide 2
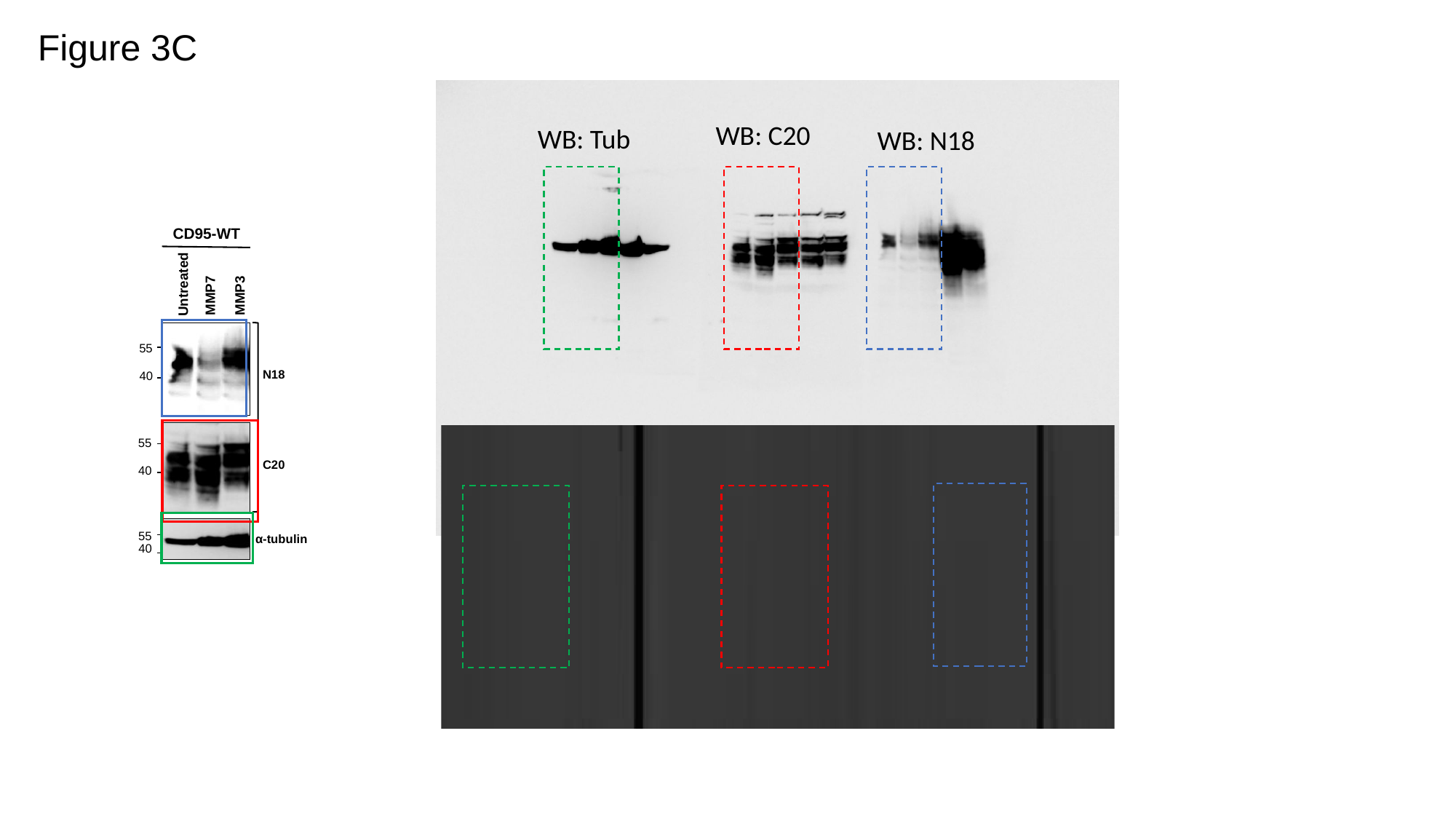

Figure 3C
WB: C20
WB: Tub
WB: N18
CD95-WT
MMP7
MMP3
Untreated
55
N18
40
55
C20
40
55
 α-tubulin
40

## Slide 3
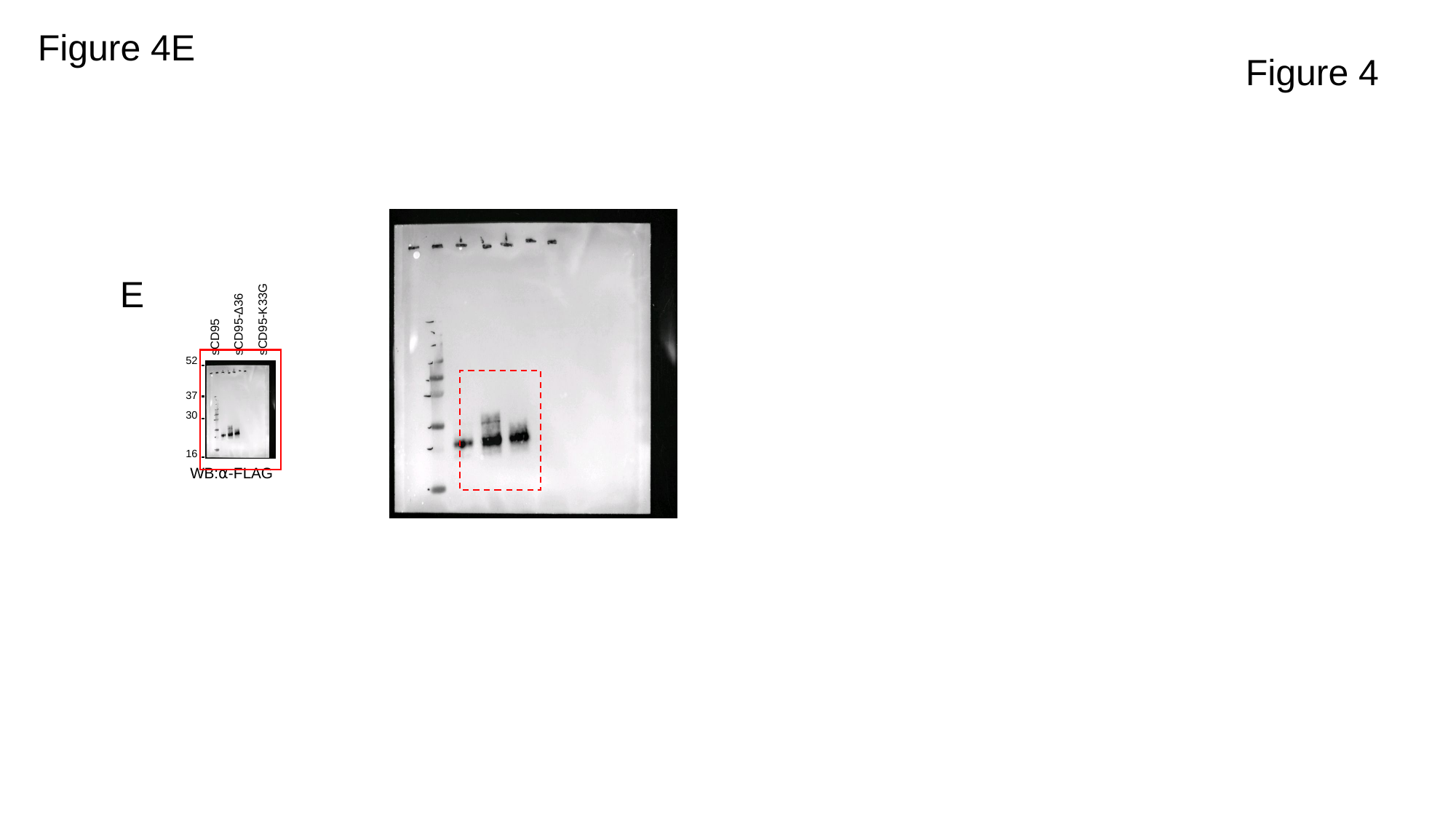

Figure 4E
Figure 4
E
sCD95-K33G
sCD95-∆36
sCD95
52
37
30
16
WB:⍺-FLAG

## Slide 4
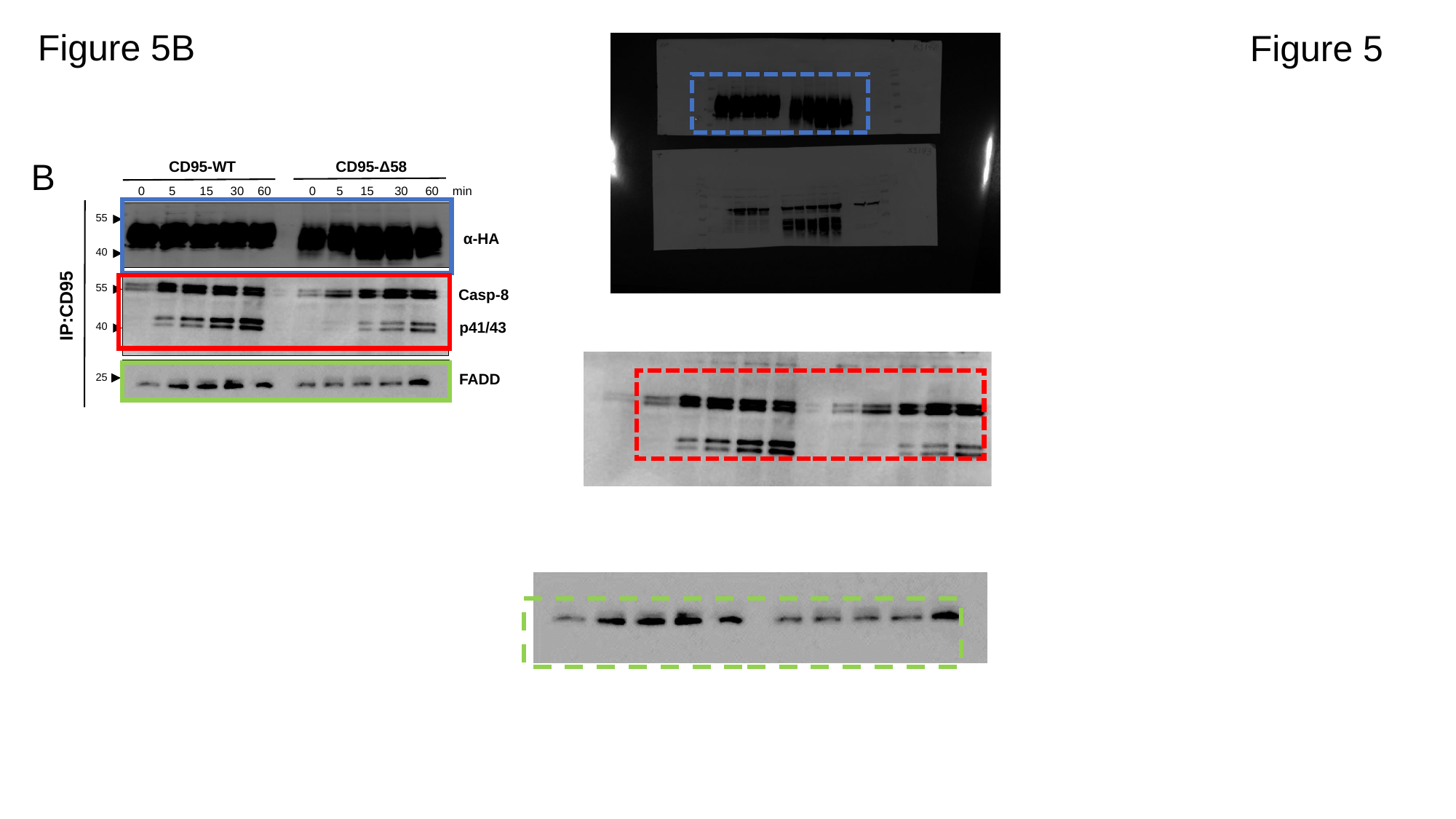

Figure 5B
Figure 5
B
CD95-WT
CD95-Δ58
0 5 15 30 60 0 5 15 30 60 min
55
 α-HA
40
55
 Casp-8
IP:CD95
p41/43
40
FADD
25

## Slide 5
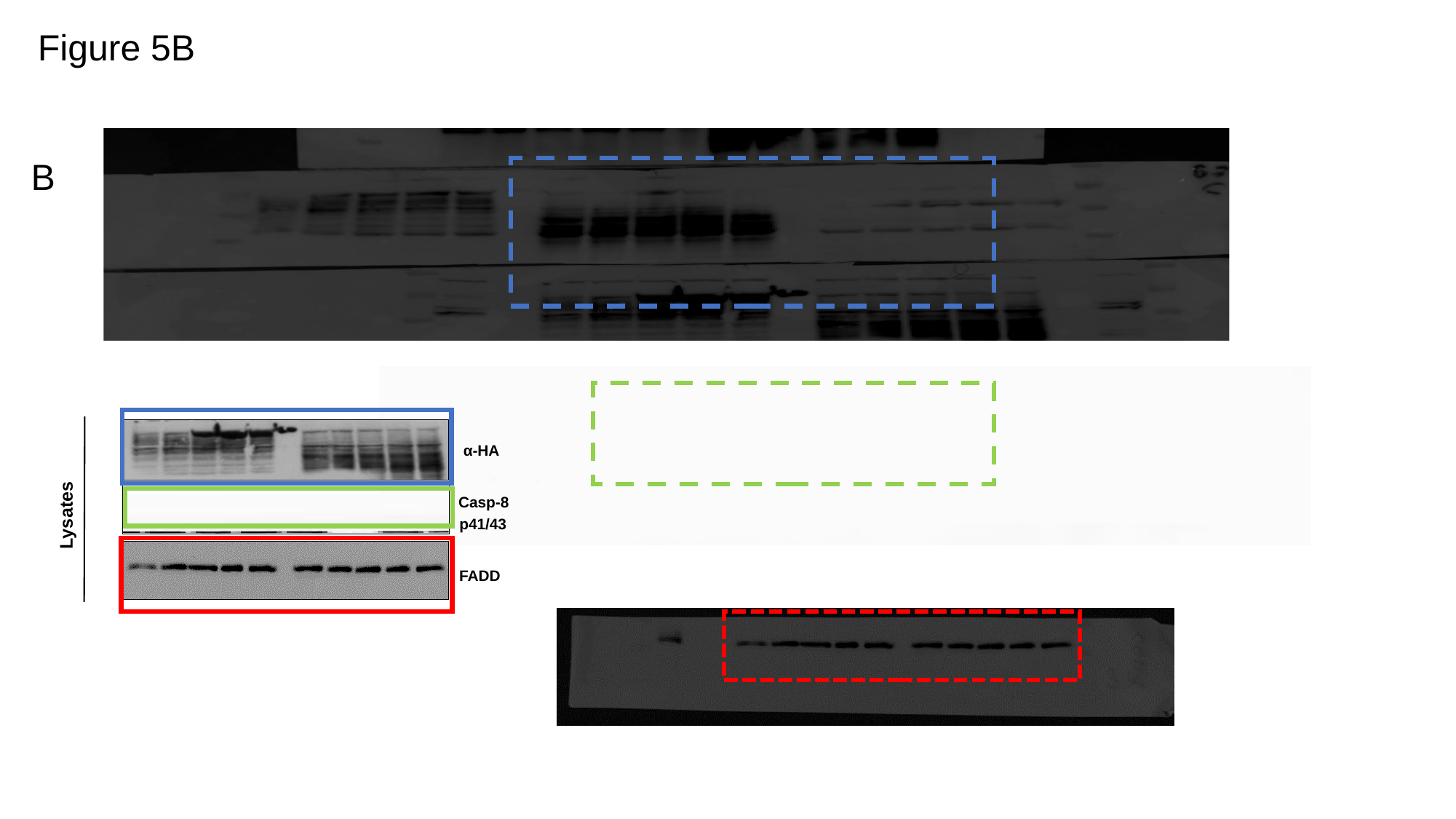

Figure 5B
B
 α-HA
 Casp-8
Lysates
p41/43
FADD

## Slide 6
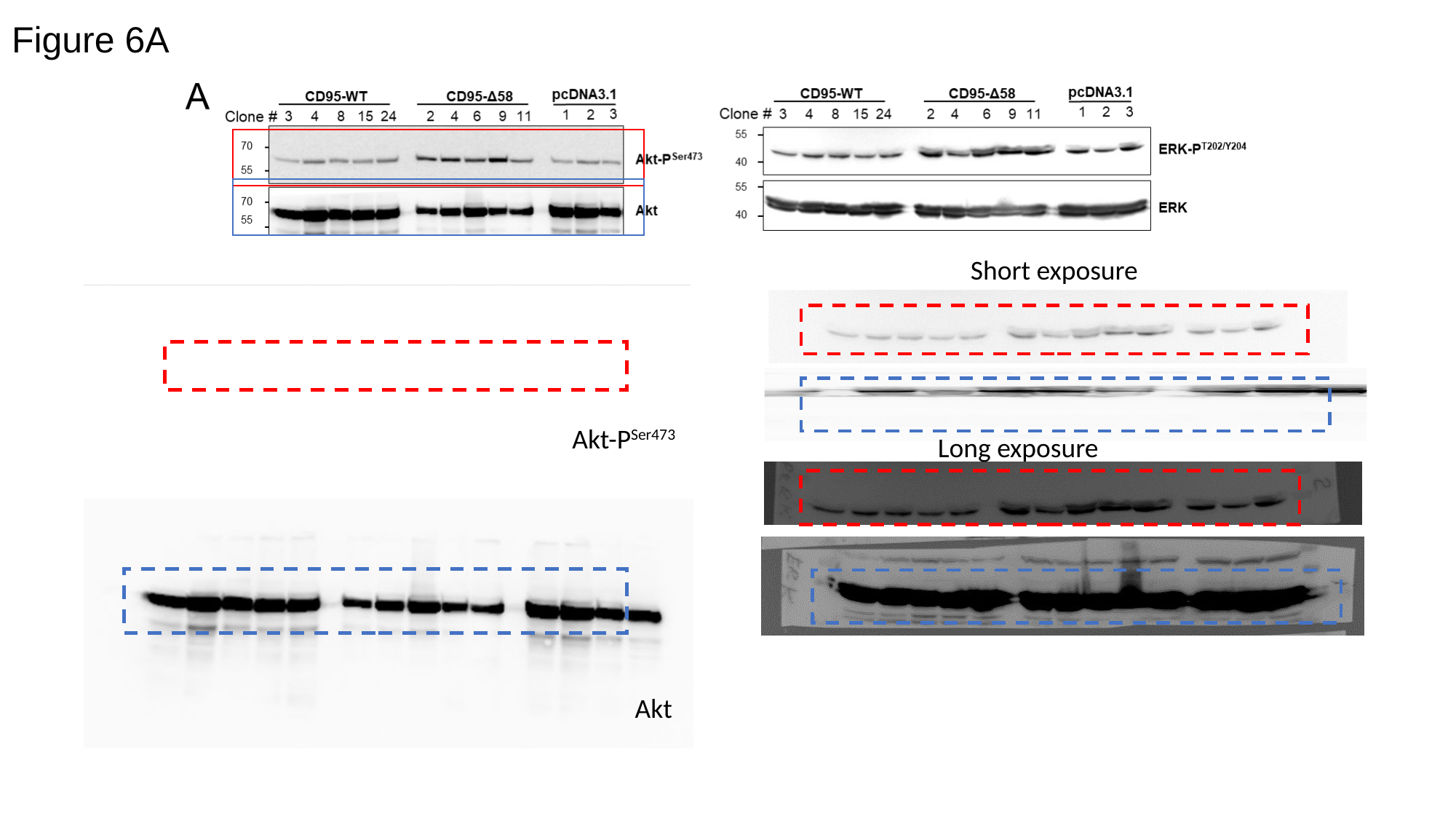

Figure 6A
Short exposure
Akt-PSer473
Long exposure
Akt

## Slide 7
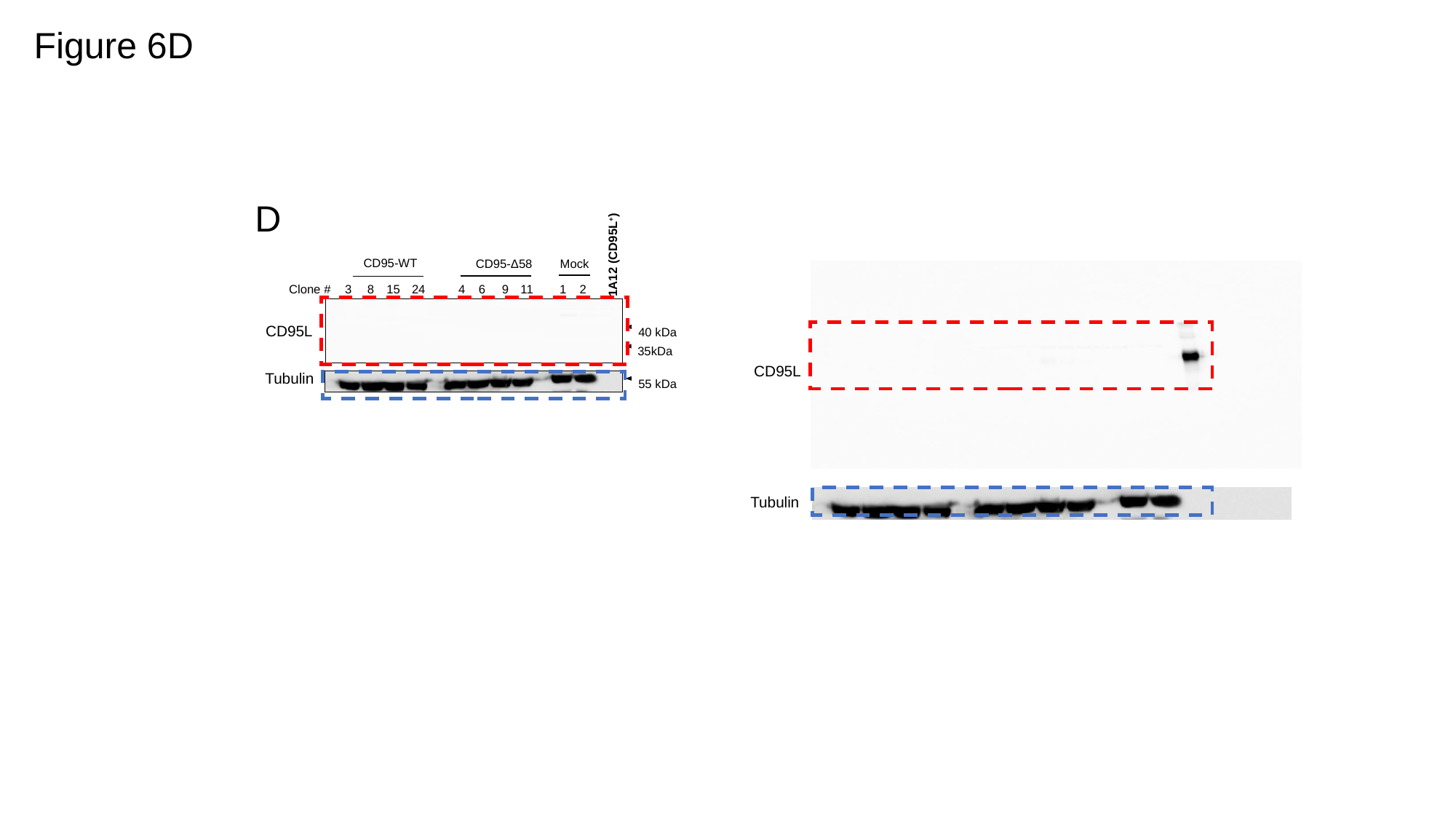

Figure 6D
D
1A12 (CD95L+)
CD95-WT
CD95-Δ58
Mock
3
8
15
24
4
6
9
11
1
2
Clone #
CD95L
40 kDa
35kDa
CD95L
Tubulin
55 kDa
Tubulin

## Slide 8
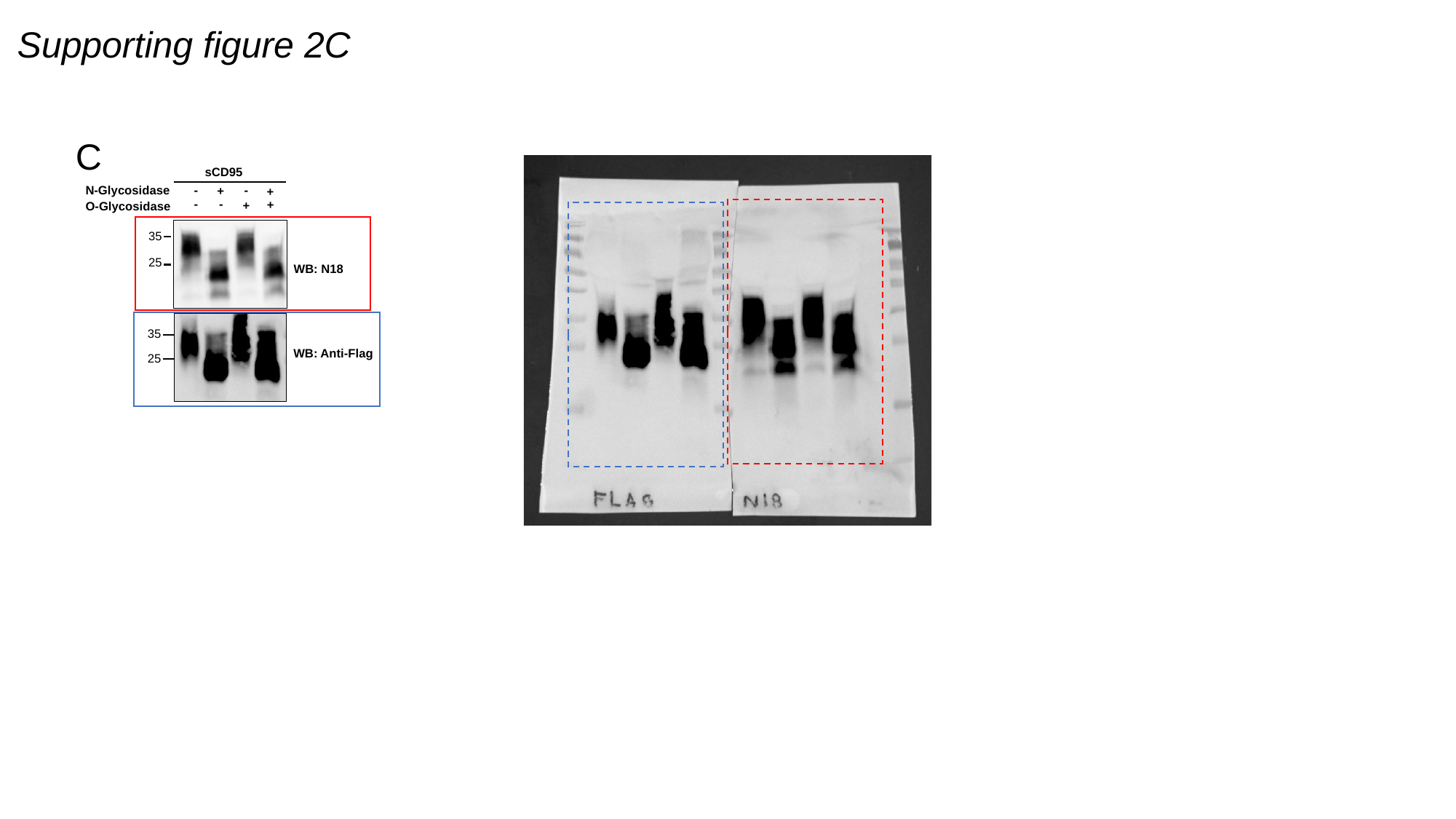

Supporting figure 2C
C
sCD95
-
-
+
+
-
-
+
+
N-Glycosidase
O-Glycosidase
35
25
WB: N18
35
WB: Anti-Flag
25

## Slide 9
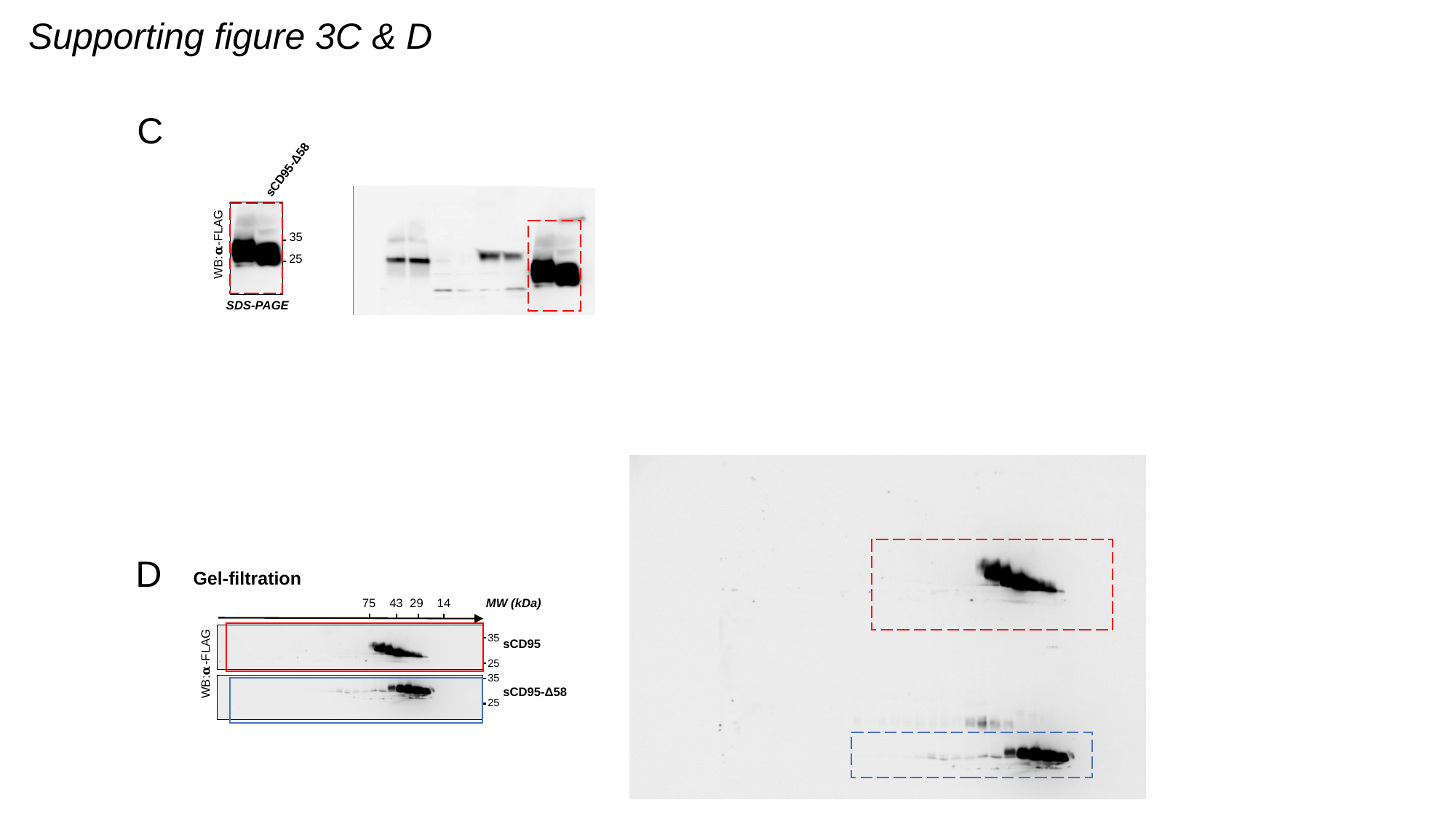

Supporting figure 3C & D
C
sCD95-Δ58
35
WB:𝛂-FLAG
25
SDS-PAGE
D
Gel-filtration
MW (kDa)
75 43 29 14
sCD95
35
WB:𝛂-FLAG
25
35
sCD95-Δ58
25
